# Beta-endoproteolysis of the cellular prion protein by dipeptidyl peptidase-4 and fibroblast activation protein

**DOI:** 10.1073/pnas.2209815120

**Published:** 2022-12-27

**Authors:** Andrew R. Castle, Sang-Gyun Kang, Ghazaleh Eskandari-Sedighi, Serene Wohlgemuth, My-Anh Nguyen, Daniel J. Drucker, Erin E. Mulvihill, David Westaway

**Affiliations:** ^a^Centre for Prions and Protein Folding Diseases, University of Alberta, Edmonton, AB T6G 2M8, Canada; ^b^Department of Medicine, University of Alberta, Edmonton, AB T6G 2G3, Canada; ^c^Department of Biochemistry, University of Alberta, Edmonton, AB T6G 2H7, Canada; ^d^University of Ottawa Heart Institute, Ottawa, ON K1Y 4W7, Canada; ^e^Department of Biochemistry, Microbiology and Immunology, Faculty of Medicine, University of Ottawa, Ottawa, ON K1H 8M5, Canada; ^f^Lunenfeld-Tanenbaum Research Institute, Mt. Sinai Hospital, Toronto, ON M5G 1X5, Canada; ^g^Department of Medicine, University of Toronto, Toronto, ON M5S 2J7, Canada

**Keywords:** beta-cleavage, dipeptidyl peptidase, DPP4, prion disease, prolyl endopeptidase FAP

## Abstract

The cellular prion protein (PrP^C^) converts to alternatively folded pathogenic conformations (PrP^Sc^) in prion infections and binds neurotoxic oligomers formed by amyloid-β α-synuclein, and tau. β-Endoproteolysis, which splits PrP^C^ into N- and C-terminal fragments (N2 and C2, respectively), is of interest because a protease-resistant, C2-sized fragment (C2^Sc^) accumulates in the brain during prion infections, seemingly comprising the majority of PrP^Sc^ at disease endpoint in mice. However, candidates for the underlying proteolytic mechanism(s) remain unconfirmed in vivo. Here, a cell-based screen of protease inhibitors unexpectedly linked type II membrane proteins of the S9B serine peptidase subfamily to PrP^C^ β-cleavage. Overexpression experiments in cells and assays with recombinant proteins confirmed that fibroblast activation protein (FAP) and its paralog, dipeptidyl peptidase-4 (DPP4), cleave directly at multiple sites within PrP^C^’s N-terminal domain. For wild-type mouse and human PrP^C^ substrates expressed in cells, the rank orders of activity were human FAP ~ mouse FAP > mouse DPP4 > human DPP4 and human FAP > mouse FAP > mouse DPP4 >> human DPP4, respectively. C2 levels relative to total PrP^C^ were reduced in several tissues from FAP-null mice, and, while knockout of DPP4 lacked an analogous effect, the combined DPP4/FAP inhibitor linagliptin, but not the FAP-specific inhibitor SP-13786, reduced C2^Sc^ and total PrP^Sc^ levels in two murine cell-based models of prion infections. Thus, the net activity of the S9B peptidases FAP and DPP4 and their cognate inhibitors/modulators affect the physiology and pathogenic potential of PrP^C^.

## Significance

In the fatal brain disorders known as prion diseases, the cellular prion protein (PrP^C^) is converted into an abnormal structure by other abnormal prion protein molecules. A fragmentation process known as β-cleavage that splits PrP^C^ into two parts is associated with prion diseases, but a clear description of the underlying cleavage mechanism is lacking. Here, we use cultured cells, cell-free systems, and mouse models to show that β-cleavage of PrP^C^ can be performed by two proteins closely related to each other: dipeptidyl peptidase-4 and fibroblast activation protein. By applying inhibitors of these proteins to prion-infected cells, we also show that the β-cleavage activity of dipeptidyl peptidase-4 in particular may be important in the pathogenesis of prion diseases.

**Fig. 1.**
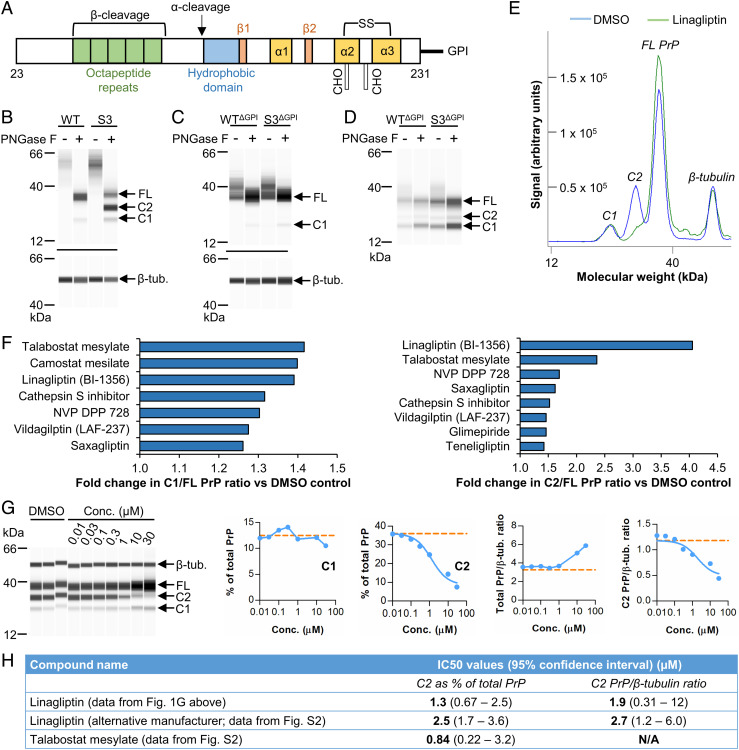
Modulation of S3 PrP fragmentation studied with mutants and protease inhibitors. (*A*) Structural features of mature PrP^C^ (residues 23 to 231), including the α-helices and β-strands, disulfide (SS) bond, and sites for attachment of N-linked glycans (denoted as CHO). Approximate cleavage sites are also marked. (*B*–*D*) Capillary western data showing PrP immunodetection (Sha31 antibody) in PNGase F-treated RK13 cell lysates (*B*, and *C*) or conditioned media samples (*D*) obtained following transient expression of the indicated PrP variants. (*E*) Example capillary western data (Sha31 antibody; +PNGase F) comparing chemiluminescence spectra from DMSO- and linagliptin-treated S3-3 RK13 cells. (*F*) Modulators of S3 PrP fragmentation identified from a compound library screen in S3-3 RK13 cells (4 d treatment at 20 µM). (*G*) Capillary western data (Sha31 antibody; +PNGase F) showing that linagliptin treatment dose-dependently reduces C2 PrP levels in S3-3 RK13 cells (orange line = mean DMSO control value). Curves were fitted by linear regression for the plots showing C2 data. (*H*) Table summarizing IC_50_ values obtained for linagliptin and talabostat. Also refer to *SI Appendix*, Fig. S2. β-tub., beta-tubulin; conc., concentration.

The cellular prion protein (PrP^C^) is a glycoprotein that can misfold into a protease-resistant form (PrP^Sc^), a process integral to the pathogenesis of prion diseases. Although most abundant on the cell surface of neurons, normally folded PrP^C^ is expressed by various cell types (1), and its central region is subject to distinct fragmentation events (highlighted in Fig. 1*A*). The major cleavage fragment in healthy brain tissue, known as C1, is the C-terminal product of α-cleavage, which is thought to occur within residues 108 to 111 (2, 3) and was first ascribed to a disintegrin and metalloproteinases (ADAMs), particularly ADAM10 (4–7), although other studies have questioned this link (8–11). Conversely, in prion-infected brains, full-length (FL) PrP^Sc^ can be converted to a truncated form consisting of residues ~90 to 231 (2), either as a result of endoproteolytic cleavage by calpains (12) or N-terminal trimming by lysosomal cathepsins up to the beginning of the protease-resistant misfolded domain (13, 14). The truncated form of PrP^Sc^, originally referred to as C2, accumulates as disease progresses (2, 12, 15, 16), potentially becoming more abundant than FL PrP^Sc^ by the terminal stage (17). The truncated PrP^Sc^ is also very similar in size to the core of FL PrP^Sc^ (sometimes called PrP27–30) that is resistant to proteinase K (PK) digestion (18), a common in vitro method for detecting the presence of misfolded PrP. Later, the term “β-cleavage” was introduced to describe the generation of similarly sized “C2” fragments from normally folded PrP^C^ in uninfected cells (19), with several studies outlining a Cu^2+^-dependent hydrolysis mechanism mediated by reactive oxygen species (ROS), potentially resulting in cleavage of cell-surface PrP^C^ at multiple sites within the octarepeat (OR) domain and the stretch of Gly residues that follow OR5 (6, 20–22); however, such a mechanism has never been demonstrated in vivo. To avoid confusion, hereafter we refer to the PrP^C^ β-cleavage product as C2 and the truncated form of PrP^Sc^ as C2^Sc^.

Clarifying the mechanisms responsible for the central region cleavages is important for a number of reasons: i) unlike the C2 fragment, C1 is resistant to misfolding and is a dominant-negative inhibitor of PrP^Sc^ formation (23, 24); ii) the abundant C2^Sc^ in prion-infected brains could be generated either from truncation of FL PrP^Sc^ or misfolding of C2 produced by β-cleavage of PrP^C^, and the relative contributions of these two processes are unclear; iii) PrP^C^ is reportedly a receptor for soluble aggregates of proteins associated with other neurodegenerative disorders (amyloid-β, α-synuclein, and tau) and acts as a mediator of their neurotoxicity (25), but the putative binding sites for these aggregates are absent in C1, while the central region site remains intact in C2; iv) although found only at minimal levels in the healthy brain, C2 can make up the majority of PrP^C^ detected in certain tissues (26–28), which suggests that β-cleavage is of physiological importance.

Here, using small-molecule compounds, capillary western analysis of PrP fragmentation (29), and reversed genetics, two type II membrane proteins of the S9B peptidase subfamily were found to perform β-endoproteolysis of PrP^C^: fibroblast activation protein (FAP; also known as prolyl endopeptidase FAP), which is associated with tissue remodeling (30), and dipeptidyl peptidase-4 (DPP4; also known as CD26), an established drug target in type 2 diabetes (31). While internal cleavage of PrP^C^ holoprotein substrates—mapped both to OR and pre-OR regions—was not anticipated from the typical cleavage site preferences of these proteases, these functional relationships indicate possibilities for altering the production of PrP substrates that drive neurodegeneration.

## Results

### Modulation of S3 PrP Fragmentation Studied with Mutants and Protease Inhibitors.

The “S3” PrP variant displays accentuated β-cleavage in the rabbit kidney epithelial cell line RK13 and in the brains of transgenic mice due to missense mutations introduced into the OR domain (32); S3 PrP thus comprises a tool to study β-cleavage. First, we investigated whether this cleavage occurs at the cell surface. Previous analyses had shown that S3 PrP is expressed at the cell surface and is then trafficked to internal compartments in a similar manner to wild-type (WT) PrP (32). Here, we altered S3 PrP by introducing a stop codon to preclude glycophosphatidylinositol (GPI) anchor addition (S3^ΔGPI^ PrP) and used a corresponding WT PrP variant as a control [WT^ΔGPI^ PrP; (33)]. Unlike unaltered S3 PrP with its normal membrane attachment and high C2 production (Fig. 1*B*), C2 was undetectable when S3^ΔGPI ^PrP was transiently expressed in RK13 cells (Fig. 1*C*). Because anchorless PrP forms are secreted, we analyzed conditioned media and again found similar fragmentation patterns for WT^ΔGPI^ and S3^ΔGPI^ PrPs (Fig. 1*D*). The importance of the GPI anchor for PrP^C^ β-cleavage suggests that C2 production can occur at the cell surface and, indirectly, that the corresponding protease is present at the same location, which expands upon previous findings obtained using a surface biotinylation approach (21).

To identify putative endoproteases, we screened 130 protease inhibitors at 20 µM concentration using RK13 cells stably expressing S3 PrP (clone 3, hereafter referred to as S3-3). Effects on PrP^C^ fragmentation were assessed using a higher-throughput PNGase F digestion protocol followed by capillary western analysis, noting a mild, systematic overestimation of fragment sizes using this method (29). Nine compounds were considered hits, because they decreased the C1/FL PrP or C2/FL PrP ratios (or both) by >1.25-fold without causing excessive toxicity (Fig. 1 *E* and *F* and Dataset S1), which was defined as a reduction of >50% in levels of the internal loading control β-tubulin. Camostat, a broad-spectrum inhibitor of trypsin-like serine proteases, was the only compound to reduce the C1/FL PrP ratio specifically. However, given the large number of trypsin-like serine proteases, we decided to focus here on the putative β-cleavage inhibitors, because the eight compounds that reduced the C2/FL PrP ratio mostly consisted of inhibitors of specific proteases—six targeting DPP4 and one targeting cathepsin S. Cathepsins have previously been linked to PrP^Sc^ truncation (13, 14), but we were unable to replicate the initial screening data when the cathepsin S inhibitor was re-tested in S3-3 RK13 cells under the same conditions (*SI Appendix*, Fig. S1). In contrast, testing dose ranges of the DPP4 inhibitors in S3-3 RK13 cells confirmed that all six compounds were able to reduce C2 levels relative to total PrP (Fig. 1*G* and *SI Appendix*, Fig. S2). However, because the DPP4 inhibitors tended to increase total PrP expression at high concentrations, only linagliptin consistently reduced C2 levels relative to β-tubulin. Testing linagliptin from two different sources produced highly similar results, with half-maximal inhibitory concentrations (IC_50_s) of 1.3 to 2.7 µM being obtained (Fig. 1*H*). Switching to calculating C1 and C2 levels relative to total PrP, rather than relative to FL PrP, confirmed that the effects of the DPP4 inhibitors were largely specific to the C2 fragment, with minimal changes to C1 levels observed (Fig. 1*G* and *SI Appendix*, Fig. S2). Additional DPP4 inhibitors that did not reduce the C2/FL PrP ratio in the library screen were similarly inactive upon re-testing (*SI Appendix*, Fig. S1).

DPP4 removes X-Pro/Ala N-terminal dipeptides (where X is any amino acid) from substrates of <100 residues, including glucagon-like peptide-1 and amyloid-β peptides (31, 34), as long as the third residue is not Pro (i.e., X-Pro/Ala↓Pro). Internal cleavage of PrP^C^, a protein of >200 residues, is not in line with the canonical function of DPP4, but certain DPP4 inhibitors target FAP as well; indeed, the most effective compounds in our study, linagliptin and talabostat, reportedly inhibit FAP with IC_50_s of 370 nM and 70 nM, respectively, which are closer to the values we observed than the relatively consistent low-nanomolar potencies that the DPP4 inhibitors display toward DPP4 itself (34, 35). Moreover, in addition to its DPP4-like exopeptidase activity, FAP acts as an endopeptidase favoring the sequence Gly–Pro↓Pro (30, 36, 37)—motifs present in multiple copies within the mutated S3 PrP OR domain (Gly–Gly–Gly/Ser→Gly–Pro–Gly/Ser). Further clues pointing in the direction of FAP were as follows: i) FAP expression was detected in RK13 cells but was absent from several cell lines that do not display enhanced C2 levels when S3 PrP is expressed (*SI Appendix*, Fig. S3) (32); and ii) the enhanced β-cleavage of S3 PrP was almost matched by a single G86P substitution in the WT OR domain, but a G86P.G87P double mutant (with a Pro residue now immediately C-terminal of the hypothetical cleavage site) lost this ability (*SI Appendix*, Fig. S4*A*). Thus, we concluded that FAP was most likely responsible for the high levels of S3 PrP β-cleavage in RK13 cells.

### Expression of DPP4 or FAP Increases Relative C2 PrP Levels in Cultured Cells.

**Fig. 2.**
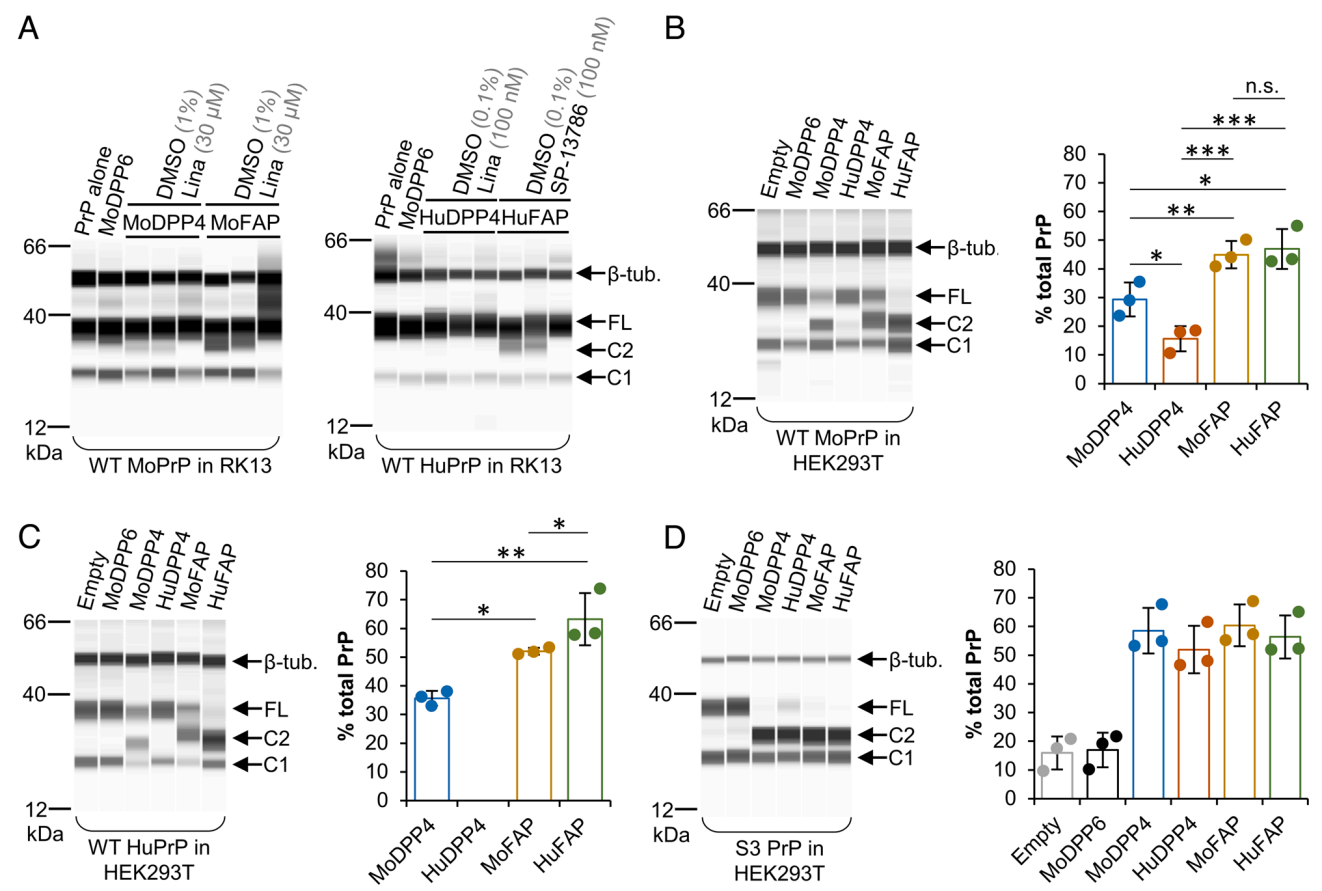
Expression of DPP4 or FAP increases relative C2 PrP levels in cultured cells. (*A*) Representative capillary western data (+PNGase F) from RK13 cells transiently co-expressing the indicated S9B peptidases and WT MoPrP or WT HuPrP (n = 3 and n = 2 independent experiments, respectively). (*B*–*D*) Representative capillary western data (+PNGase F) from HEK293T cells transiently co-expressing the indicated S9B peptidases with the indicated form of PrP. The empty vector condition (pcDNA3) was the control for HuDPP4 and HuFAP; an exact empty vector control for MoDPP4 and MoFAP was unavailable. Charts show the mean C2 levels (as % of total PrP) ± S.D. (n = 3 independent experiments; one-way ANOVA and Newman–Keuls multiple comparisons test; *P < 0.05; **P < 0.01; ***P < 0.001). Absence of a bar indicates that a clearly distinguishable C2 peak was not obtained. Pairwise comparisons between the different DPP4 and FAP groups in (*D*) were non-significant in all cases (*P* > 0.05). Sha31 anti-PrP antibody was used for all panels. Also refer to *SI Appendix*, Fig. S5. Lina., linagliptin; n.s., not significant.

Although FAP favors Gly–Pro motifs, cleavage activity after Gly and Ser has been reported in vitro (36), suggesting that FAP-mediated β-cleavage of WT PrP^C^ could take place. To test this possibility, we first performed transient transfection experiments in the parental RK13 cell line, which lacks detectable endogenous PrP^C^ expression. C2 was undetectable by capillary western analysis when WT mouse or human PrPs (MoPrP, HuPrP) were expressed either on their own or together with dipeptidyl peptidase-6 (DPP6)—a catalytically dead member of the same family as DPP4 and FAP; however, co-expression with FAP of the equivalent species resulted in a C2 band (Fig. 2*A*). In spite of its reported lack of endopeptidase activity, mouse DPP4 (MoDPP4) also induced a C2 band when co-expressed with WT MoPrP, although co-expression of HuPrP and human DPP4 (HuDPP4) did not result in detectable levels of C2 production (Fig. 2*A*). Linagliptin and the FAP-specific inhibitor SP-13786 blocked DPP4- and FAP-mediated increases in C2 levels, respectively, when tested at doses as low as 100 nM, whereas the vehicle control treatment (dimethyl sulfoxide, DMSO) had no effect (Fig. 2*A*). In contrast, neither MoDPP4- nor MoFAP-mediated C2 production was affected by various inhibitors of unrelated proteases, including calpain, lysosomal, and metalloproteinase inhibitors (*SI Appendix*, Fig. S5 *B* and *C*). Thus, we concluded that DPP4 and FAP increase C2 levels by acting on PrP^C^ directly rather than by regulating activity of downstream proteases (including those previously linked to PrP cleavage processes).

The disparity between MoDPP4 and HuDPP4 in terms of their ability to induce PrP^C^ β-cleavage led us to perform further co-transfection experiments in the human cell line HEK293T. FAP of either species was somewhat more efficient at cleaving both MoPrP and HuPrP than MoDPP4, with the HuPrP–HuFAP combination resulting in the highest cleavage efficiency (Fig. 2 *B* and *C*). HuFAP also generated C2 fragments from the endogenous PrP^C^ expressed by HEK293T cells (*SI Appendix*, Fig. S5*D*). As was the case in RK13 cells, co-expression of HuPrP and HuDPP4 in HEK293T cells resulted in negligible C2 levels, although, surprisingly, co-expression of MoPrP and HuDPP4 did produce a faint C2 band (Fig. 2 *B* and *C*). Since the expression of S3 PrP in HEK cell derivatives does not result in the same baseline enhancement to β-cleavage that is observed in RK13 cells (32), we co-expressed the proteases with S3 PrP in HEK293T cells. Interestingly, all the proteases, including HuDPP4, induced similarly high levels of C2 production from S3 PrP (Fig. 2*D*). Finally, human dipeptidyl peptidases 8 and 9 (HuDPP8, HuDPP9), members of the S9B peptidase subfamily that are expressed in the cytoplasm (38), were tested as controls; neither HuDPP8 nor HuDPP9 induced β-cleavage of HuPrP (*SI Appendix*, Fig. S5*G*), as could be predicted from the largely cell-surface expression pattern of PrP^C^ (39). Together, these observations from co-transfection experiments provided firm support for the unexpected involvement of S9B peptidases in PrP^C^ β-cleavage.

### Identification of DPP4 and FAP Cleavage Sites in the PrP^C^ N-Terminal Domain.

**Fig. 3.**
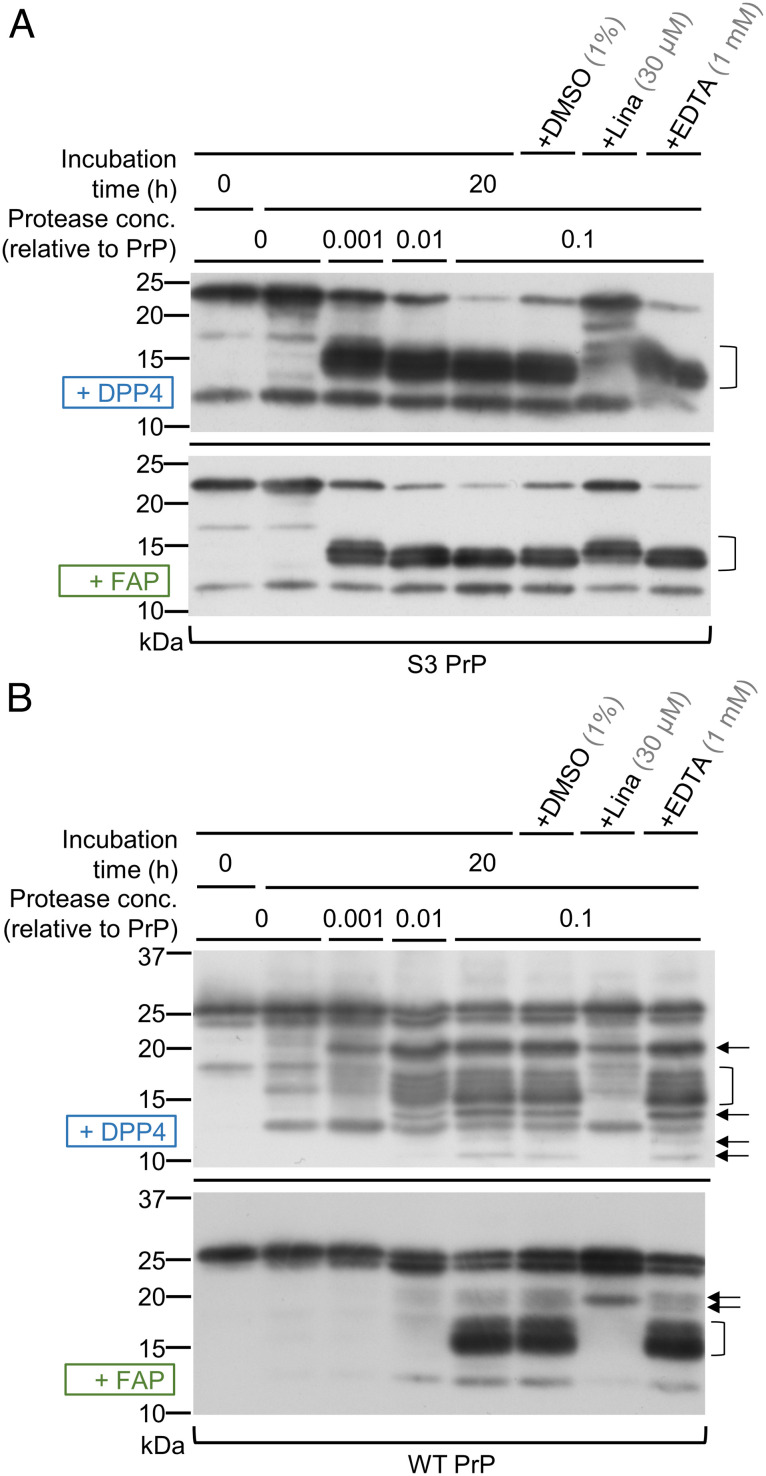
RecDPP4 and recFAP cleave recS3PrP and recWTPrP at multiple positions. (*A* and *B*) Immunoblots (Sha31 antibody) showing the fragmentation patterns observed following incubation of recS3PrP or recWTPrP with recDPP4 or recFAP. Protease concentrations are expressed relative to the PrP concentration (which is given an arbitrary value of 1.0). C2-sized fragments generated by the proteases are indicated by brackets and bands of different sizes by arrows.

To assess direct effects of DPP4 and FAP upon PrP^C^ substrate, we incubated recombinant S3 or WT PrP (recS3PrP, recWTPrP) with recombinant DPP4 or FAP (recDPP4, recFAP) for 20 h at 37 °C (mouse sequences in all cases). Conventional Western blotting was used for these experiments to obtain better resolution of similarly sized, low-molecular weight (MW) bands (<20 kDa) than was achievable by capillary western. We found that each protease generated multiple C2-sized fragments from both forms of recPrP (Fig. 3 *A* and *B*), although higher relative amounts of protease were required to process recWTPrP. Fragmentation of recWTPrP by recDPP4 was prevented by linagliptin doses as low as 300 nM (except for the production of a ~20 kDa band), and fragmentation by recFAP was partially blocked by 3 µM linagliptin (*SI Appendix*, Fig. S6*A*). Furthermore, addition of EDTA (ethylene diamine tetra acetic acid;1 mM) or use of broad-spectrum inhibitors of cysteine, aspartic acid, and metalloproteases at 10 µM (E64d, pepstatin A, and marimastat, respectively) had no effects (Fig. 3 *A* and *B* and *SI Appendix*, Fig. S6*B*), ruling out that (hypothetical) contamination of the recDPP4 and recFAP preparations by metal ions [leading to ROS-dependent hydrolysis (20, 21)] or by other proteases was generating the observed cleavages.

**Fig. 4.**
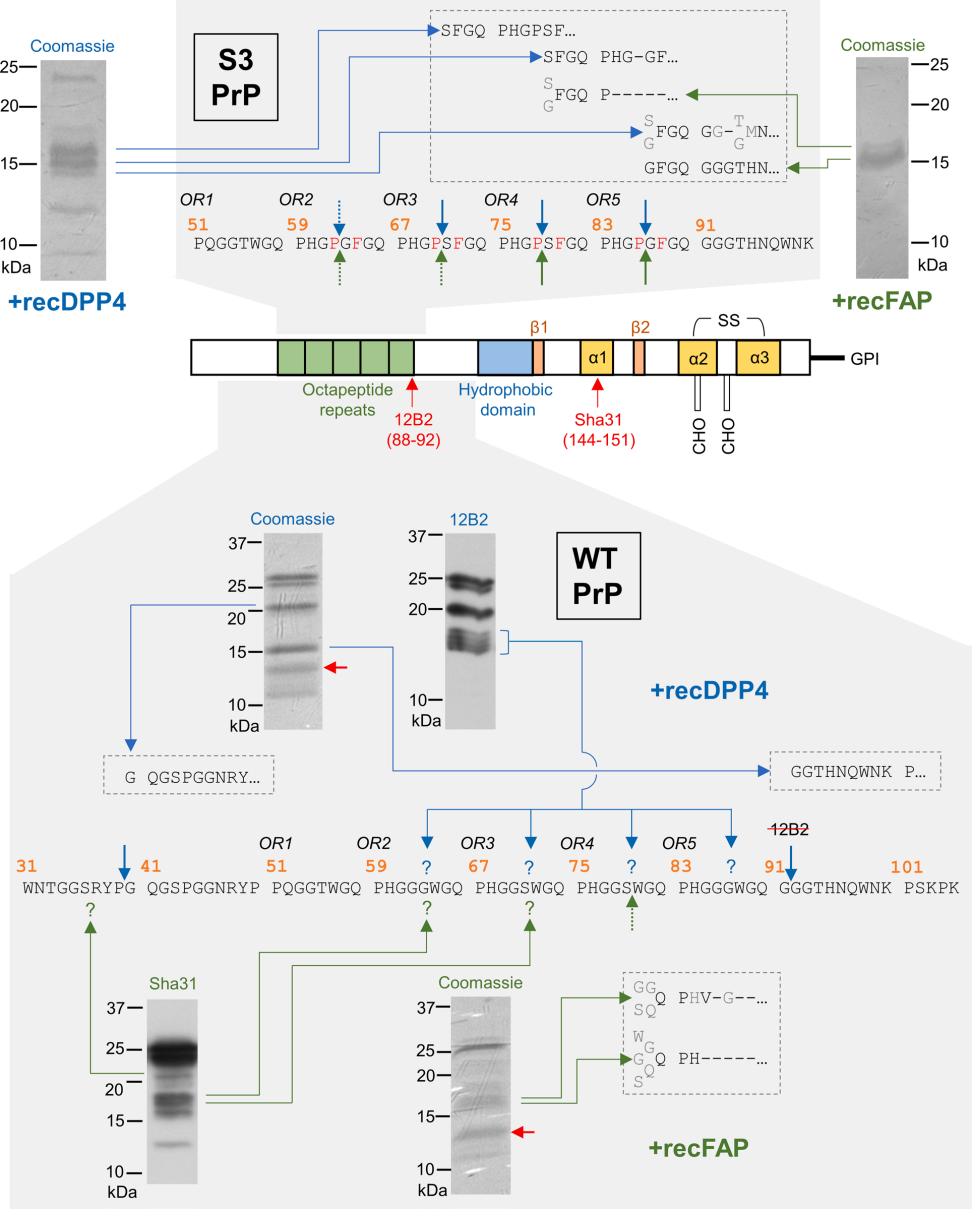
Confirmed and probable DPP4 and FAP cleavage sites in the PrP^C^ N-terminal domain based upon multiple pieces of data, including Edman sequencing of Coomassie-stained bands (protease concentration was 1/1,000 of the PrP concentration for S3 PrP and 1/10 for WT PrP). For ease of understanding, Lane 4 from *SI Appendix*, Fig. S6*D* (+DPP4; 12B2 antibody), and Lane 4 from *SI Appendix*, Fig. S8 (+FAP; Sha31 antibody), are recapitulated here. Antibody epitopes are indicated on the diagrammatic representation of PrP^C^. Red arrows denote bands also present in the absence of protease. Edman sequencing reads are shown in the boxes and are aligned to the PrP sequence. Low-confidence identifications, including when multiple possible residues were identified, are shown in gray. Dashes indicate that a specific amino acid could not be identified. Solid blue and green arrows denote confirmed DPP4 and FAP cleavage sites, respectively. Dotted arrows denote likely cleavage sites, and question marks indicate additional potential cleavage sites.

Having established endoproteolytic scission of recPrP by recDPP4 and recFAP, we assigned cleavage site residues by N-terminal Edman sequencing. The starting material for this technique consisted of bands excised from Coomassie-stained membranes, accounting for minor discrepancies from immunoblotting band patterns. We found that recDPP4 and recFAP cleaved recS3PrP between adjacent Pro and Ser/Gly residues within the OR domain (Fig. 4); as mentioned previously, these Pro residues in the S3 allele replace Gly residues present in the WT prototype. Due to the need for higher amounts of protease for fragmentation and the requirement for several hundred nanograms of each fragment for Edman analysis, it was not possible to sequence all the fragments generated from recWTPrP. Nonetheless, several definite and potential cleavage sites were identified. First, the ~13 kDa band typically present both in protease-containing and control reactions (Figs. 3 and 4) consisted of two fragments starting at residues 113 and 116 that were most likely spurious products of proteolysis occurring during recPrP expression in *E. coli* (40). Second, unexpected processing close to the N terminus was observed—after Pro39 for recDPP4 (~20 kDa band) and at two nearby sites for recFAP that could not mapped to the level of individual residues (Fig. 4). Given that FAP favors Gly in the P2 position (30, 36), it is unlikely to cleave after Pro39 (Tyr in P2); one potential alternative cleavage is after Ser36. Intact N-terminal fragments resulting from these cleavage events were not detectable (*SI Appendix*, Fig. S6*C*), perhaps due to disruption of the EB8 antibody epitope (residues 26 to 34 (41)) by additional processing even closer to the N terminus. As for the other fragments produced by recDPP4, we identified a cleavage site after Gly91, which is slightly C-terminal of the OR domain (Fig. 4). RecDPP4 also most likely cleaved recWTPrP within each of ORs 2 to 5, although the exact sites remain undefined. For recFAP, combining the Edman sequencing and Western blotting data suggests that cleavages after Gly63, Ser71, and Ser79 occurred. Further support for FAP-mediated processing following OR Ser residues derives from experiments in RK13 cells in which co-expression of MoFAP with a variant PrP allele containing G70S, S71G, G78S, and S79G substitutions (G/S-switch PrP) reduced C2 levels relative to total PrP by ~50% compared to co-expression with WT MoPrP (*SI Appendix*, Fig. S4 *C*–*G*). A similar effect was observed when G/S-switch PrP was co-expressed with MoDPP4. Alignment of PrP^C^ sequences from several mammalian species indicates that the identified cleavage sites are generally well-conserved (*SI Appendix*, Fig. S7).

Since the PrP^C^ N-terminal domain is intrinsically unstructured, we considered whether the observed cleavages might result from an N- to C-terminal “scanning” (i.e., processive) mechanism of proteolytic action. The detection of recDPP4- and recFAP-generated N2 fragments—the N-terminal products of β-cleavage—with apparent MWs of ≥6 kDa argued against this hypothesis (*SI Appendix*, Fig. S6*C*). However, to prevent the putative cleavage by DPP4 after Pro39, we engineered a PrP expression construct with a G40P substitution, thereby creating a Pro–Pro motif (which blocked the enhanced C2 production resulting from a G86P substitution, as described earlier). If the cleavage mechanism was processive, then the G40P substitution should also eliminate more C-terminal cleavage events, but both DPP4 and FAP retained the ability to induce C2 production when co-expressed with G40P PrP in RK13 cells (*SI Appendix*, Fig. S4*B*). Furthermore, time course analyses of recWTPrP digestion by recDPP4 revealed that the different C2-sized bands (~14.5 to 18 kDa) were equally abundant at the earliest time point (*SI Appendix*, Fig. S8), whereas bands of higher MW would likely be more prominent at this stage if a processive cleavage mechanism was present. The lowest-MW band of this group did become progressively more intense with increasing incubation time, but this could be the result of cycles of cleavage and release that trimmed longer fragments to the minimal size. Similarly, no conclusive evidence of processivity was obtained from time course analyses of recWTPrP incubated with recFAP (*SI Appendix*, Fig. S8).

### C2 PrP Levels in *Fap*-Knockout Tissues Confirm FAP as an In Vivo β-PrPase.

**Fig. 5.**
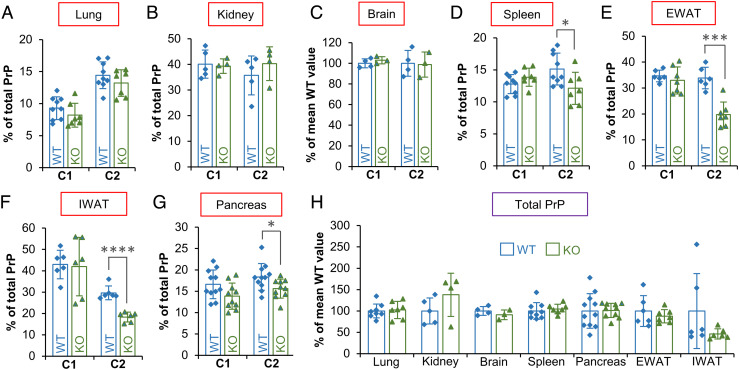
C2 PrP levels in *Fap*-KO mouse tissues confirm FAP as an in vivo β-PrPase. (*A*–*G*) Relative C1 and C2 levels in PNGase F-treated homogenized mouse tissues (WT and *Fap* KO) were determined by capillary westerns (*A*, *D*–*G*) or conventional Western blotting (*B* and *C*). For chart (*C*), fragment levels were calculated relative to the sum of the FL and C1 band signals and then normalized to the mean WT value, because the C2 bands had to be quantified from a different exposure to the FL and C1 bands. Data are shown as means ± SD (unpaired two-sample *t* tests; n = 3 to 11; *P < 0.05; ***P < 0.001; ****P < 0.0001). (*H*) Chart showing that total PrP expression levels were not significantly affected by *Fap* KO. Data are shown as means ± SD. Total PrP signals were corrected for loading error (if possible). Also refer to *SI Appendix*, Fig. S10. EWAT, epididymal white adipose; IWAT, inguinal white adipose.

We next sought evidence as to whether DPP4 and/or FAP act as β-PrPases in vivo. First, we analyzed tissues obtained from the *Dpp4*^tm1Nwa^ line of *Dpp4*-knockout (KO) mice (42). While *Dpp4* genotype had no effect on the levels of C2 relative to total PrP in homogenized lung or kidney, we observed a trend toward reduced relative C2 levels in *Dpp4*-null spleens that was close to statistical significance (*SI Appendix*, Fig. S9 *A*–*C*). Even though C2 levels in healthy brain tissue are low, the fragment was sufficiently abundant for detection and quantification by conventional Western blotting. However, *Dpp4* KO did not significantly affect brain C2 levels (*SI Appendix*, Fig. S9*D*). Similarly, relative C2 levels were not reduced in lung, kidney, or brain tissues obtained from *Fap* KO (*Fap*^em1Tcp^/Ddr) mice (43), but relative C2 levels were reduced significantly from 15.1 to 12.1% in *Fap*-null spleens (Fig. 5 *A*–*D*). Analyses of additional *Fap*-null tissues showed that relative C2 levels were around two-fifths lower in epididymal and inguinal adipose tissues (EWAT, IWAT) compared to WT controls and were reduced by a lesser extent in the *Fap*-null pancreas; in contrast, relative C1 levels and total PrP expression levels were unaffected (Figs. 5 *E*–*H*). We later observed that the variable effects of *Fap* KO seemed to reflect differing levels of FAP expression (in the WT context) among the tissue types analyzed (*SI Appendix*, Fig. S10*J*). Finally, in contrast to suggestions that β-cleavage is rare or even absent in healthy tissues (44), C2 levels were generally comparable to those of the C1 fragment, with the exception of brain tissue (Fig. 5; also refer to *SI Appendix*, Figs. S9 and S10). Considered together, these data indicate that FAP functions as a β-PrPase in vivo.

### Linagliptin Treatment Reduces PrP^Sc^ Levels in Prion-Infected Cells.

**Fig. 6.**
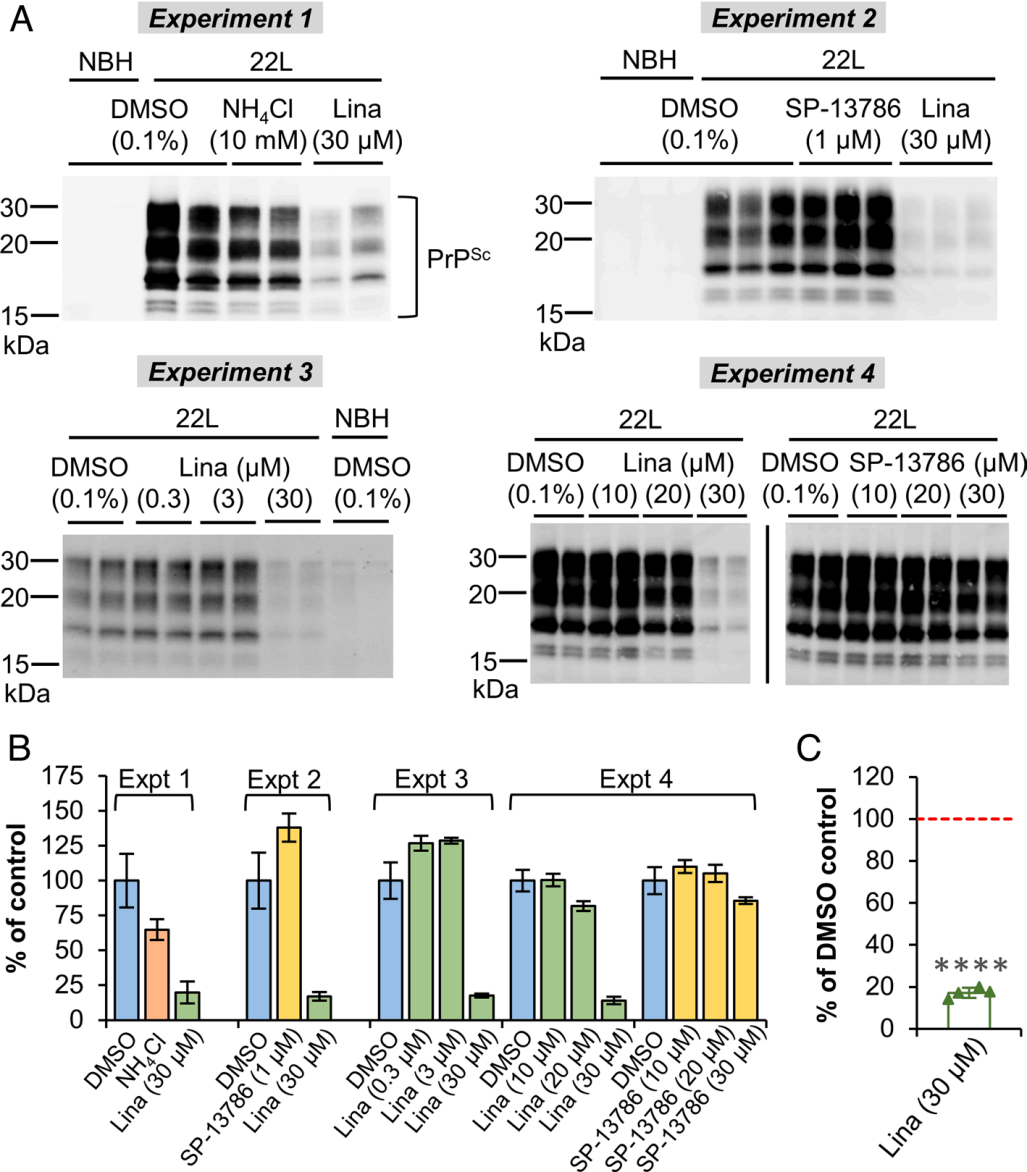
Linagliptin reduces PrP^Sc^ levels in 22L-infected primary cerebellar glial cultures. (*A*) Immunoblots (Sha31 antibody) of PK-treated lysates prepared at either 21 (experiments 1 and 2), 28 (experiment 3), or 35 DPI (experiment 4). (*B*) Chart showing quantification data (means ± SD) from the immunoblots shown in (*A*). DMSO was the vehicle control for all compound treatments. Each numbered experiment was performed using freshly prepared primary cultures (n ≥ 2 technical replicates in each case). (*C*) Chart showing the mean ± SD for 30 µM linagliptin treatment calculated from the values obtained from each independent experiment shown in (*B*) (n = 4; one-sample *t* test; ****P < 0.0001).

As previously mentioned, the abundant C2^Sc^ in prion-infected brains could be generated either from truncation of FL PrP^Sc^ or misfolding of C2 produced by β-cleavage of normally folded PrP^C^. Interestingly, work using velocity gradient fractionation of brain tissues from infected animals showed that an increase in high-MW PK-resistant assemblies including C2^Sc^ is paralleled by increased levels of C2-sized fragments in upper gradient fractions that mostly contain monomeric PrP species (16). Although undetermined whether or not the C2-sized fragments in upper gradient fractions are protease-sensitive, these data hint at the possibility that β-cleavage of PrP^C^ could be up-regulated in infected brain tissue. We therefore investigated whether inhibition of DPP4/FAP-driven β-cleavage would reduce total PrP^Sc^ accumulation using two cell culture models of prion replication: murine C2C12 myoblast cells differentiated into post-mitotic myotubes (45, 46) and primary cerebellar glial cultures (47). In C2C12 myotubes exposed to Rocky Mountain Laboratory (RML) prions (a mouse-adapted isolate originally derived from the prion disease scrapie), linagliptin (30 µM) consistently reduced PK-resistant PrP^Sc^ levels by 32 to 37% at 5, 10, and 20 days post-infection (DPI), as assessed by conventional Western blotting (*SI Appendix*, Fig. S11). Furthermore, in cerebellar glial cultures infected with 22L prions (another mouse-adapted scrapie strain) and harvested at 21 to 35 DPI, linagliptin (30 µM) reduced PK-resistant PrP^Sc^ levels by 83%, on average (Fig. 6). Lower doses of linagliptin and doses of the FAP-specific inhibitor SP-13786 up to 30 µM had little impact on accumulation of PK-resistant PrP^Sc^ in these cultures, while 10 mM NH_4_Cl resulted in a moderate reduction of 35% (Fig. 6).

In addition to analyzing PK-resistant PrP^Sc^, we assessed the sum of PK-resistant C2^Sc^ plus PK-sensitive C2 by PNGase F treatment of lysates from the above experiments. Summed C2 levels relative to total PrP had increased markedly in differentiated C2C12 cells by 20 DPI (*SI Appendix*, Fig. S12 *A*–*C*), thereby mimicking the situation in diseased brain tissue. However, addition of linagliptin (30 µM) had only a marginal effect, if any, on the increase in C2 abundance. In contrast, although summed C2 accumulation in the 22L-infected cerebellar glial cultures was somewhat variable between experimental replicates, linagliptin (30 µM) consistently reduced summed C2 levels relative to total PrP (*SI Appendix*, Fig. S12 *D*–*F*). Treatment with lower doses of linagliptin, doses of the FAP-specific inhibitor SP-13786 up to 30 µM, or 10 mM NH_4_Cl either had no effect on summed C2 levels relative to total PrP or resulted in only minimal reductions. Finally, although C2 levels in uninfected cerebellar glial cultures were low (thus complicating accurate quantification), linagliptin treatment also tended to reduce their levels of C2 relative to total PrP^C^ (*SI Appendix*, Fig. S12 *D*–*F*).

Overall, our inhibitor studies suggest that DPP4-mediated β-cleavage is an important driver of PrP^Sc^ accumulation in these cell-based infection models. We also observed that DPP4 appears to colocalize with PrP^C^ in (uninfected) cerebellar glial cultures and may be up-regulated by linagliptin treatment (*SI Appendix*, Fig. S13), the latter bringing to mind upregulation of plasma DPP4 following linagliptin dosing in mice (48). While reducing PK-resistant PrP^Sc^ levels required higher doses of linagliptin than those previously reported as necessary for DPP4 inhibition (49), this may reflect intrinsic differences in the paradigms including i) the use of intact cells versus cell extracts; ii) the formation of PrP^Sc^ from FL substrate in addition to formation from C2; and iii) differing mechanisms of clearance of C2^Sc^ and normally folded C2.

## Discussion

### Mechanism and Products of PrP^C^ β-Cleavage.

In this study, we demonstrate that DPP4 and FAP, both type II membrane proteins of the S9B peptidase subfamily, perform β-cleavage of the cell-surface glycoprotein PrP^C^. DPP4 and FAP share 52% sequence identity (50) and lie within 100 kb of each other on both the mouse and human versions of chromosome 2 (accession numbers listed below). While physical interactions between PrP^C^ and DPP6 were described previously (51, 52), the catalytically active members of the S9B family were not previously considered as candidate PrP^C^ β-PrPases due to their canonical cleavage specificities. Also, earlier studies had linked β-cleavage of PrP^C^ to the metalloproteinase ADAM8 (6) as well as to a non-enzymatic, Cu^2+^-mediated hydrolysis mechanism involving ROS (20–22), while PK-resistant C2^Sc^ fragments (with N termini around residue 90) were thought to be generated from FL PrP^Sc^ in prion-infected brain tissue by lysosomal cathepsins (13, 14) or calpains (12). However, while concentrations of DMSO (a hydroxyl radical quencher) as low as 5 mM virtually eliminated the C2 production induced by serum deprivation of SH-SY5Y cells (21), we found here that the increase in C2 levels resulting from transfection with DPP4 or FAP was unaffected by exposure to 1% (v/v) DMSO (~141 mM) or to calpain, lysosomal, or metalloproteinase inhibitors, arguing against indirect effects of DPP4 and FAP on the mechanisms noted above. Indeed, in vitro experiments confirmed that recDPP4 and recFAP can cleave recPrPs by direct enzymatic action. While endopeptidase activity has been ascribed previously to FAP (30), DPP4 is regarded solely as an aminopeptidase (31). Interestingly, DPP4 was reported to display endopeptidase activity, specifically gelatinase activity (53), but this finding was later contradicted (54–56); our data align with the original conclusion.

Rather than a unique β-site, processing of recWTPrP by recFAP appeared to take place at GGG↓WGQ and GGS↓WGQ sequences in ORs 2 to 4. Although FAP preferentially cuts after Gly-Pro motifs (30), in vitro cleavages following Gly or Ser have been reported (36). Furthermore, Gly may be favored in the P3 position (37), which could explain why MoFAP was less efficient at cleaving G/S-switch PrP (PHGGSWGQ→PHGSGWGQ) than WT MoPrP. Cleavage of recWTPrP by recDPP4 seemed to occur within each of ORs 2 to 5, possibly at the same positions as FAP, given that the G/S-switch substitutions had a similar effect on MoDPP4-mediated C2 production. Interestingly, recWTPrP was also cleaved by recDPP4 following Gly91, which is part of the degenerate repeat that follows OR5; this position differs from an earlier assignment of cleavage after Asn96 (19) but would generate C2 fragments similar in size to the C2^Sc^ that accumulates in prion-infected brain tissue (2, 12, 15–17).

Surprisingly, processing of recWTPrP by recDPP4 and recFAP also occurred close to the N terminus at conserved sites in the vicinity of hexarepeat 1 (57). This finding is intriguing given that DPP4 and FAP are firmly associated with neuropeptide biogenesis (58–61) and that residues 23 to 33 of PrP^C^ determine activation of the G-protein-coupled receptor Adgrg6 (62). It is also notable that a similar N-terminal fragment (23–41) was detected in the brain of an individual who died of Gerstmann–Straussler–Schienker syndrome, a human prion disorder (63). However, the intrinsically unstructured PrP^C^ N-terminal domain may be constrained in terms of its conformations in vivo by the presence of interacting proteins, which could mean that not all the DPP4 and FAP cleavage sites identified in these in vitro experiments are replicated in vivo. N-terminomic ligase methods (64) using ex vivo tissue samples may be a useful approach to confirm (or refute) whether a metabolically stable neuropeptide can, in fact, be generated from PrP^C^.

### Physiology and Pathophysiology of PrP^C^ β-Cleavage.

Analysis of tissues from *Fap*-null mice confirmed that FAP is involved in PrP^C^ β-cleavage in vivo. *Fap* KO had a variable effect on C2 levels in the different tissues analyzed, which can be attributed partly to differing levels of FAP expression (in the WT context); for example, *Fap* KO did not affect C2 levels in the brain, in which FAP was undetectable by capillary western regardless of genotype. However, DPP4 may be at least partly responsible for C2 production in the brain, because we observed that the DPP4/FAP inhibitor linagliptin reduced PK-resistant PrP^Sc^ and summed C2 levels (PK-resistant plus PK-sensitive) in prion-infected primary cerebellar glial cultures (brain-derived cells), whereas FAP-specific inhibition had little or no effect. Although these data seemingly diverge from the lack of effect of *Dpp4* KO on relative C2 levels in the healthy brain, an attractive model, albeit a speculative one, is that interference effects from endogenous serine protease inhibitors (65) could limit DPP4-mediated β-cleavage under normal conditions, with this inhibition then being released in the presence of neuroinflammation. In this regard, it is notable that inflammation up-regulates astrocytic DPP4 expression (66) and that conversion of astrocytes to a neuroinflammatory or “reactive” phenotype appears to be an important early step in prion disease pathogenesis (67, 68). Of course, while this model can explain how DPP4 inhibition could reduce C2^Sc^ levels specifically, a reduction in total PrP^Sc^ would not necessarily be expected, unless there is a difference in conversion efficiency between C2 and FL PrP^C^ substrates—something that remains to be determined.

In contrast to DPP4, FAP may act as a tissue-specific regulator of PrP^C^ β-cleavage outside of a prion disease context, perhaps affecting the physiological function of PrP^C^. Indeed, in opposition to suggestions that β-cleavage is much rarer than α-cleavage in healthy tissues (44), we have shown that most mouse tissues, with the exception of brain, contain similar levels and C2 and C1 fragments, and this finding complements recent studies demonstrating abundant β-cleavage in pancreatic β cells and multiple cell types of the eye (26–28, 69). Interestingly, PrP^C^ may play a role in endothelial-to-mesenchymal transitions of trabecular meshwork cells in the eye, a cell type with high levels of C2 relative to total PrP (27). PrP^C^ has also been linked to epithelial-to-mesenchymal transitions in cell culture models (70). Both types of transition are sources of cancer-associated fibroblasts, which typically express high levels of FAP (71). Of course, even in tissues in which *Fap* KO significantly reduced C2 fragment levels, residual β-cleavage activity was still present. Analysis of mouse models lacking both FAP and DPP4 expression would help to exclude (or otherwise) the possibility of a compensatory increase in DPP4-mediated β-cleavage activity in the absence of FAP, although a double-KO mouse model cannot be generated simply through crosses of the preexisting single-KO lines due to the tight linkage of *Dpp4* and *Fap* on chromosome 2. Furthermore, some proportion of β-cleavage may be mediated by previously described mechanisms (i.e., ADAM8 and/or Cu^2+^-dependent hydrolysis involving ROS). If one or both of these mechanisms are confirmed to exist in vivo, then the nomenclature of PrP^C^ fragmentation events may need refining to account for the presence of multiple β-cleavage-like mechanisms.

Returning to the prion disease context, since DPP4-mediated β-cleavage of PrP^C^ seems to promote PrP^Sc^ accumulation in primary cerebellar glial cultures, brain-penetrant DPP4 inhibitors could be tested in animal models of prion infections. Such investigations will be informative, because co-transfection experiments indicated that HuDPP4 is not an effective β-PrPase, unlike MoDPP4. If DPP4 is found to play an important role in the pathogenesis of prion disorders in mice, it would raise questions over extrapolating results from mouse models of prion infections to humans, in which DPP4-driven β-cleavage appears less likely. In contrast to HuDPP4, HuFAP efficiently cleaved HuPrP in cells, indicating that the S9B peptidase family is still connected to β-cleavage in humans; however, it remains to be seen whether FAP has any involvement in natural or iatrogenic prion diseases.

In conclusion, we demonstrate through in vitro and cell-based experiments that two type II membrane proteins—one typically associated with dipeptide processing—perform β-site endoproteolytic cleavages of PrP^C^ in the natively unstructured region. Although certain aspects of the roles that these S9B peptidases play in PrP physiology and pathophysiology will require further clarification, our results suggest possibilities for therapeutic intervention against prion diseases and axes of investigation, given the published biologies of DPP4 and FAP in neuropeptide processing (58–61), cellular senescence (72–74), and tissue remodeling (75, 76).

## Materials and Methods

Extended materials and methods can be found in *SI Appendix*, including manufacturer/supplier details for resources used in the study (*SI Appendix*, Table S2).

### Transfections.

RK13 and HEK293T cells were seeded into 6-well or 96-well tissue culture-treated plates in low-glucose Dulbecco's modified Eagle medium (DMEM) containing 10% fetal bovine serum (FBS) and 1% penicillin-streptomycin solution (pen-strep). After 24 h recovery, transient transfections were performed in duplicate using Lipofectamine 3000. If required, small-molecule compounds were also added to the culture medium at this time. The culture medium was exchanged ~6 h after transfection for fresh medium (low-glucose DMEM, 10% FBS, and 1% pen-strep) containing compounds (if required). Cells were lysed 48 h after transfection (*SI Appendix*).

### Compound Screening.

RK13 cells were seeded into 96-well tissue culture-treated microplates at 5,000 cells/well in low-glucose DMEM containing 5% FBS and 1% pen-strep. After 24 h recovery, compounds from the DiscoveryProbe Protease Inhibitor Library were added in duplicate to the culture medium. Cells were exposed to the compounds for 4 d before lysis (with fresh media containing compounds added halfway through the incubation period).

### In Vitro Protease Assays.

RecWTPrP consisting of residues 23 to 231 of murine PrP^C^ (*Prnp^b^* allele) fused to an N-terminal His-tag (MGSSHHHHHHSSGLVPRGSHML) was provided as a gift by the laboratory of Valerie Sim (77). RecS3PrP produced as part of another study (78) was provided as a gift by Nathalie Daude and consisted of residues 23 to 231 of murine PrP^C^ (*Prnp^a^* allele) with the following substitutions: G62P, W64F, G70P, W72F, G78P, W80F, G86P, and W88F. Purified recPrPs were transferred into a buffer of 25 mM Tris, pH 8, using Amicon Ultra-4 Centrifugal Filter Units. RecPrPs were incubated for varying time periods at 37 °C in the presence of murine recDPP4 or recFAP. Small-molecule inhibitors were included in certain reactions as indicated in figure legends.

### Tissue Homogenization.

The procedures used to generate the *Dpp4*^–/–^ and *Fap^–/–^* mice have been described in previous publications (42, 43). Refer to *SI Appendix*, including *SI Appendix*, Table S1, for more information about these mouse models, details of the tissues analyzed here, and the homogenization procedure used.

### Prion Infection Experiments.

Primary cerebellar glial cultures were prepared as previously described (47), although further details are provided in the *SI Appendix*. C2C12 myoblasts were differentiated into myotubes and reserve cells by switching to a culture medium of low-glucose DMEM, 10% (v/v) horse serum, and 1% pen-strep. Differentiated C2C12 cells and primary cerebellar glial cultures were exposed to brain homogenates from terminally ill C57BL/6J mice infected either with RML prions (C2C12) or 22L prions (glia) or were exposed to uninfected brain homogenate (final concentrations of 5 μg/mL total protein in all cases); preparation of these homogenates was described previously (79). After 24 h, cells were washed twice with PBS and were maintained in fresh culture medium, with subsequent media changes performed twice per week. From 3 (C2C12) or 5 DPI onwards (glia), small-molecule compounds were included in the culture medium. Lysates were prepared at various time points post-infection and were treated either with PNGase F (*SI Appendix*) or PK (50 µg/mL for 30 min).

### Capillary Western Assays.

Assays were performed using Wes or Jess capillary western instruments as described in detail in a previous publication (29). Refer to *SI Appendix*, Table S2, for details of the kits used. Note that the separation time in the protocol was changed from the default 25 to 30 min when the target protein was PrP^C^. The associated Compass software was used to generate artificial lane view images from the chemiluminescence spectra, with visual adjustment performed using the in-built contrast slider. Peak areas obtained using the default Gaussian fit were the first choice for quantification of immunostaining, but the alternative “dropped lines” fitting method was used if Gaussian fitting was inadequate. Total protein signals were calculated by summing the area values derived from the dropped lines fitting method for all the peaks detected within the 1 to 250 kDa analysis range.

### SDS-PAGE and Conventional Western Blotting.

Assays were performed largely as described in a previous publication (29). Details of immunostaining procedures used for specific primary antibodies and protocol adjustments used when collecting samples for Edman sequencing are provided in the *SI Appendix*. Densitometric analyses and adjustment of image brightness/contrast settings for display purposes were carried out using ImageJ (https://imagej.nih.gov/ij/). Briefly, individual lanes were identified with rectangular selections, and commands available in the Gels submenu were used to generate densitometric plots. Baselines were drawn manually to enclose each peak, thus enabling area values to be obtained.

### Gene Linkage Groups.

Linkage groups for the DPP4 and FAP genes on mouse and human versions of chromosome 2 are defined in mouse genome annotation release 109, assembly GCF_000001635.27 and in human genome annotation release 109.20210514, assembly GCF_000001405.39.

### Charts, Plots, and Statistical Tests.

Plots of compound dose-responses were created using GraphPad Prism. Data points were connected by straight lines unless the figure legend specifies that a dose-response curve was fitted. In such cases, the default dose-response (inhibition) model available in GraphPad Prism was used. The least square fitting method was selected, the Hill Slope constrained to –1.0, and the top of the curve constrained to the mean value obtained from vehicle control samples. IC_50_s and 95% CIs are reported to two significant figures.

All column charts were prepared using Excel, and they display means ± SD in addition to individual data points when appropriate. Statistical tests were performed in Excel or GraphPad Prism. T tests were either one-sample or two-sample (unpaired) depending on the data format. In the case of two-sample *t* tests, the heteroscedastic form was used for n < 6. For n ≥ 6, an F-test for equal variances was used to determine whether or not the homoscedastic form could be used. For groups of n ≥ 7, the Shapiro–Wilk normality test was applied; if significance divergence from normality was present, the Mann–Whitney *U* test was used instead of a *t* test. When more than two groups were compared simultaneously, one-way ANOVA was used followed by the Newman–Keuls multiple comparisons test.

## Supplementary Material

Appendix 01 (PDF)

Dataset S01 (XLSX)

## Data Availability

All study data are included in the article and/or *SI Appendix*. New materials generated for these studies are available upon request from the corresponding author.
