# Supplementary material for: Beta-endoproteolysis of the cellular prion protein by dipeptidyl peptidase-4 and fibroblast activation protein
Source: Proc Natl Acad Sci U S A. 2022 Dec 27;120(1):e2209815120. doi: 10.1073/pnas.2209815120 (PMC9910601; doi:10.1073/pnas.2209815120)
Supplement: Supplementary file 1 — Appendix 01 (PDF) [file pnas.2209815120.sapp.pdf]

PNAS

Supplementary Information for
Beta-endoproteolysis of the cellular prion protein by dipeptidyl peptidase-4 and fibroblast activation protein.

Andrew R. Castle, Sang-Gyun Kang, Ghazaleh Eskandari-Sedighi, Serene Wohlgemuth, My-Anh Nguyen, Daniel J. Drucker, Erin E. Mulvihill, and David Westaway

Corresponding author: David Westaway
Email: david.westaway@ualberta.ca

This PDF file includes:

Extended materials and methods
Figures S1 to S13
Tables S1 and S2
Legend for Dataset S1

Other supplementary material for this manuscript consists of the following:

Dataset S1

Extended materials and methods:

Please note that manufacturer/supplier details for resources used in the study can be found in Table S2 contained within this file.

Cell lines. C2C12, RK13, HEK293 and HEK293T cell lines were maintained in low-glucose DMEM supplemented with 5% (C2C12) or 10% (v/v; RK13, HEK293 and HEK293T) FBS and 1% (v/v) pen-strep. Production of RK13 cells stably expressing the S3 variant of murine PrP (clone S3-3) has been described previously (1). All cell lines were grown at 37 °C in 5% CO_2 at 95% humidity. Lysates of N2a and SH-SY5Y cell lines were provided as gifts by the laboratory of Dr. Satyabrata Kar.

DNA manipulations. Plasmids in the pcDNA3 background that drive expression of WT, S3 and $WT^{\Delta GPI}$ (WT sequence with a stop codon in position 232 to prevent glycophosphatidylinositol anchor addition) forms of murine PrP and those in the pBud background that drive expression of DPP6 and the S3.F88W and G86P forms of murine PrP had already been created for earlier studies (2-4). The human PrP expression construct (pcDNA3.huPrP) was generated by PCR amplification of the *PRNP* open reading frame from genomic DNA (from S.W.). The $S3^{\Delta GPI}$ PrP expression construct was created by ligating together gel-extracted *Pci*I-digested fragments of the S3 and $WT^{\Delta GPI}$ PrP constructs. The G/S-switch PrP expression construct was created using a synthetic *Prnp* fragment (Integrated DNA Technologies) containing G70S, S71G, G78S and S79G substitutions with *Age*I and *Xba*I restrictions sites on the 5' and 3' ends, respectively; this fragment was subcloned into the WT PrP construct that had been digested with *Age*I and *Xba*I to remove the corresponding WT sequence of *Prnp*. The G40P PrP expression construct was generated in a similar manner, this time using *Hind*III and *Bsu*36I. Finally, a Gibson Assembly Cloning Kit was used to construct the G86P.G87P PrP expression vector from a *Hind*III and *Xba*I-digested pBud.GFP fragment and a synthetic *Prnp* fragment (Integrated DNA Technologies) containing the G86P and G87P mutations. Sanger sequencing of new constructs was performed by staff at the Molecular Biology Service Unit of the Department of Biological Sciences, University of Alberta. The following expression constructs were purchased: pCMV-Sport6.moDPP4, pCMV-Sport6.moFAP, pcDNA3.1.huDPP4, pcDNA3.1.huDPP8, pcDNA3.1.huDPP9 and pcDNA3.1.huFAP (see Table S2 for details). In all plasmids, transgene expression was under control of the CMV promoter. Plasmids were purified for downstream transfection experiments using an EndoFree Plasmid Maxi Kit.

Preparation of primary cerebellar glial cultures. The University of Alberta institutional ethics review approved the protocol for preparing primary cerebellar glial cultures from mice (AUP00000356). Briefly, cells were extracted from cerebella of euthanized WT FVB/NJ mice at postnatal day 7 by mechanical and enzymatic dissociation. Cells were seeded at a density of 2×10^5 cells/cm^2 into plates coated with 10 µg/mL poly-D-lysine hydrobromide and were incubated at 37 °C in 5% CO_2 at 95% humidity. Cells were cultured in MEM containing 10% FBS, 25 mM KCl and 1% pen-strep. A media change was performed after 24 h with subsequent changes twice per week. Once the cultures reached confluence (after ~3 weeks), the cells were trypsinized and seeded at 1×10^6 cells/well into poly-D-lysine-coated, 6-well, tissue culture-treated microplates or onto microscope cover glass.

Immunofluorescence staining and imaging. Cells were fixed by incubation in 4% (v/v) paraformaldehyde (pH 7.4) for 15 min and were permeabilized in PBS containing 0.1% (v/v) Triton X-100 (PBST). After blocking for 30 min in 1% (w/v) BSA in PBST containing 300 mM glycine, the cells were incubated overnight at 4 °C with primary antibodies diluted in blocking buffer. Secondary antibodies conjugated to Alexa Fluor dyes (Invitrogen) were used to visualize the target molecules. Counterstaining for nuclei was performed with Hoechst 33342. Cells were imaged using an LSM700 laser scanning confocal microscope with Z-stack functions under identical imaging settings. Maximum intensity projection images were obtained using the associated Zen 2010b SP1 imaging software and pixel intensity data were obtained using Image J (https://imagej.nih.gov/ij/).

Cell lysis and collection of conditioned medium. For experiments using 96-well plates, the culture medium was aspirated and replaced with ice-cold lysis buffer consisting of 50 mM Tris, pH 7.4, 150 mM NaCl, 1% (v/v) Nonidet P-40 substitute, 0.5% (w/v) sodium deoxycholate, 0.36% (w/v) SDS, 29 mM DTT, 1 mM EDTA, and a protease inhibitor cocktail. After 15 min incubation on ice, the plate was centrifuged at 1000 × *g* for 10 min at 4 °C and supernatants were transferred either to microcentrifuge tubes or to 96-well untreated polypropylene microplates if subsequent treatment with PNGase F was required (see relevant section). For experiments using 6-well plates, the cell monolayer was rinsed twice with ice-cold PBS before lysis using the aforementioned buffer lacking DTT and with the SDS concentration adjusted to 0.1%. After 15 min incubation on ice, lysates were scraped into tubes (combining duplicates) and clarified by centrifugation at 15000 × *g* for 10 min at 4 °C. Total protein concentrations of the supernatants were determined by bicinchoninic acid assay. For experiments requiring the collection of conditioned media, the culture media from replicate wells were aspirated, combined and concentrated 6-fold using Amicon Ultra-15 Centrifugal Filter Units.

Further information on mouse models and details of the tissues used in this study. Homozygous *Dpp4*-null mice of the $Dpp4^{tm1Nwa}$ line are fertile and healthy, with no major abnormalities observed (5). However, *Dpp4*-null mice do have increased levels of insulin secretion and improved glucose tolerance (5), findings that led to the development of DPP4 inhibitors for the treatment of type II diabetes in humans. The $Fap^{em1Tcp/Ddr}$ line of *Fap*-null mice were generated more recently using CRISPR/Cas9 gene editing (6). Again, homozygous mice of this line do not display any major abnormalities. However, in contrast to the *Dpp4*-null mice, glucose tolerance and insulin secretion were unaffected by *Fap* KO (6).
Animal handling procedures and husbandry were in accordance with Canadian Council on Animal Care guidelines. Experimental procedures were approved by the Animal Care Committee at the University of Ottawa (AUPs 2909 and 2920). $Dpp4^{+/+}$ and $Dpp4^{–/–}$ littermates were housed in pairs in standard cages at 23 ˚C on a 12 h light and dark cycle. Upon sacrifice at 8–10 weeks of age, mice were perfused with ice-cold PBS (~10 mL) and tissue dissections were performed *via* midline incision. The $Fap^{+/+}$ and $Fap^{–/–}$ EWAT and IWAT as well as a portion of the pancreas tissues analysed here derive from an earlier study (6) in which mice were either fed regular chow throughout or were raised on regular chow before being switched to a 45% high-fat diet at 12 weeks of age (this dietary intervention was incidental to the aims of the current study). $Fap^{+/+}$ and $Fap^{–/–}$ littermates were sacrificed at 40 weeks of age by CO_2 asphyxiation (no PBS perfusion). EWAT were extracted from mice fed regular chow, whereas pancreas tissues and IWAT were from mice switched onto the high-fat diet. Additional $Fap^{+/+}$ and $Fap^{–/–}$ tissues (lung, kidney, spleen, brain and extra pancreas tissues) were derived from a separate colony of the same line; these mice were cared for, sacrificed, perfused with PBS and dissected as described above for the $Dpp4^{–/–}$ mice. In all cases, extracted tissues were snap-frozen on liquid nitrogen prior to storage at –80 °C. Further information on the tissues is provided in Table S1.

Tissue homogenization. Non-brain tissues were manually homogenized using a Dounce homogenizer in ice-cold lysis buffer consisting of 50 mM Tris, pH 7.4, 150 mM NaCl, 1% Nonidet P-40 substitute, 0.5% sodium deoxycholate, 0.1% SDS, 1 mM EDTA and a protease inhibitor cocktail. Homogenates were clarified by centrifugation at 15000 × *g* for 10 min at 4 °C. Total protein concentrations in the supernatants were determined by bicinchoninic acid assay. Brain tissues were processed in the same manner except that PBS containing 1 mM EDTA and the protease inhibitor cocktail was used in place of lysis buffer and the homogenates were not clarified prior to use.

PNGase F treatment. Cell lysates or tissue homogenates were deglycosylated using PNGase F when PrP^C was the target for immunoblotting or capillary western assays. All reagents used were from a New England Biolabs kit. Samples in tubes were incubated at 98 °C for 10 min in the presence of Glycoprotein Denaturing Buffer. The denatured samples were deglycosylated by 1 h incubation at 37 °C with 5–15 units/µL PNGase F (depending on total protein concentration) in the presence of GlycoBuffer 2 and 1.25% (v/v) Nonidet P-40. For cell lysates in 96-well plates, the 98 °C incubation step in the presence of Glycoprotein Denaturing Buffer was omitted.

Further immunostaining details for western blots. 5% (v/v) skimmed milk in tris-buffered saline (25 mM Tris, pH 8.0, 137 mM NaCl and 2.7 mM KCl) containing 0.1% Tween-20 (TBS/0.1%-T) was the blocking buffer and antibody diluent for 12B2 anti-PrP (7) and anti-β-tubulin primary antibodies. For the EB8 anti-PrP primary antibody (8), 5% milk in TBS/0.1%-T and 1% milk in TBS/0.1%-T were the blocking buffer and antibody diluents, respectively. 5% BSA in TBS/0.1%-T was the blocking buffer and antibody diluent for the anti-β-actin primary antibody. For the Sha31 anti-PrP primary antibody (9), the blocking step was omitted and TBS containing 0.5% Tween was the primary antibody diluent; 5% milk in TBS/0.1%-T was the secondary antibody diluent. All washes were performed with TBS/0.1%-T, except for the washes after incubation with Sha31 primary antibody (TBS containing 0.5% Tween).

Edman sequencing. Samples were denatured and reduced, separated by SDS-PAGE and transferred to membranes as previously described (1), except for the following changes: *i)* Immobilon-PSQ polyvinylidene fluoride membranes were used to enhance retention of low-MW fragments; and *ii)* a split-buffer transfer system consisting of 60 mM Tris, 40 mM N-cyclohexyl-3-aminopropanesulfonic acid, pH 10, and either 15% (v/v) ethanol (anode) or 0.1% SDS (cathode) was used to minimize glycine contamination. After transfer, membranes were rinsed once with H_2O, incubated for 1 min in Coomassie stain (0.3% [w/v] Coomassie Brilliant Blue R-250, 45% [v/v] methanol, and 10% [v/v] glacial acetic acid), and were destained by several 5 min washes in 50% methanol. Membranes were air-dried and the bands of interest excised using a razor blade. Edman sequencing was performed by staff at The Protein Facility of the Iowa State University Office of Biotechnology. Briefly, each membrane fragment was loaded into a Shimadzu PPSQ-53A instrument and subjected to 10 cycles of Edman degradation. A Wakopak Wakosil PTH-GR column was used with a flow rate of 300 µL/min and a gradient elution approach. The major labelled amino acid in each cycle was determined by manual inspection of the absorbance spectrum (269 nm) by staff at the facility.

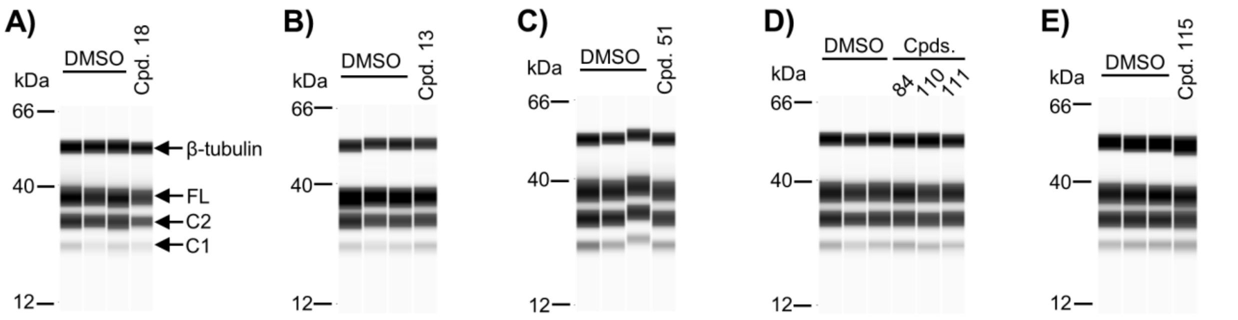

F)

Cpd. #	Compound name	Initial screen (20 µM)		Rescreening (20 µM)	
		FC in C1/FL PrP ratio	*FC in C2/FL PrP ratio*	*FC in C1/FL PrP ratio*	*FC in C2/FL PrP ratio*
18	Cathepsin S inhibitor	-1.32	-1.52	+1.18	-1.11
13	Alogliptin (SYR-322)	1.00	-1.23	+1.44	+1.17
51	Trelagliptin	+1.05	+1.07	+1.17	1.00
84	Sitagliptin phosphate monohydrate	1.00	+1.07	+1.06	-1.10
110	MK3102	+1.14	+1.13	+1.20	+1.05
111	Sitagliptin (phosphate)	+1.04	+1.18	+1.02	+1.01
115	Alogliptin benzoate	+1.12	+1.21	+1.31	1.00

Fig. S1. Data from re-screening of several compounds from the protease inhibitor library. **(A–E)** Capillary western images (Sha31 antibody; +PNGase F) showing the effects on PrP fragmentation when several protease inhibitors were re-tested using S3-3 RK13 cells (20 µM for 4 d). “DMSO” indicates exposure solely to vehicle (1% [v/v] DMSO). All compounds except #18 were DPP4 inhibitors that did not reduce the C2/FL PrP ratio by >1.25-fold in the initial library screen. **(F)** Table summarizing the data from initial testing and re-testing. Fold changes are expressed in comparison to the mean DMSO control value. New abbreviations: Cpd., compound; FC, fold change.

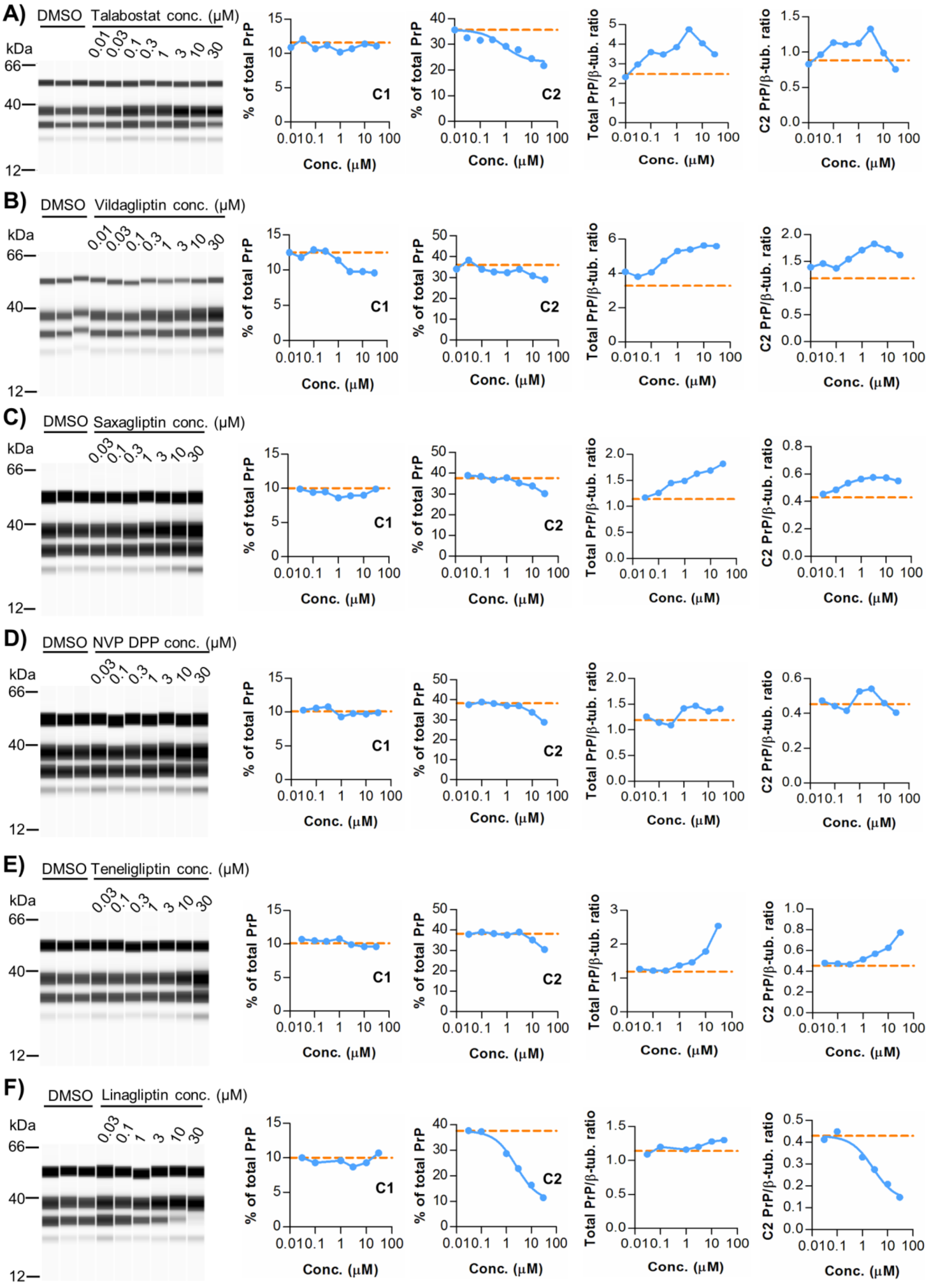

A)
DMSO
Talabostat conc. (μM)
B)
DMSO
Vildagliptin conc. (μM)
C)
DMSO
Saxagliptin conc. (μM)
D)
DMSO
NVP DPP conc. (μM)
E)
DMSO
Teneligliptin conc. (μM)
F)
DMSO
Linagliptin conc. (μM)
kDa
66
40
12
% of total PrP
Total PrP/β-tub. ratio
C2 PrP/β-tub. ratio
Conc. (μM)
C1
C2

Fig. S2 (previous page). Capillary western images (Sha31 antibody; +PNGase F) and scatter plots showing the effects of **(A)** talabostat, **(B)** vildagliptin, **(C)** saxagliptin, **(D)** NVP DPP 728, **(E)**, teneligliptin, and **(F)** linagliptin on PrP fragmentation in S3-3 RK13 cells (orange line = mean DMSO control value). Linagliptin was sourced from Toronto Research Chemicals (as opposed to the ApexBio version used in Fig. 2). From high to low apparent MW, the four bands correspond to β-tubulin, FL PrP, C2 and C1. Data points are connected by straight lines except for the plots in **(A & F)** showing C2 levels as % of total PrP and the plot in **(F)** showing C2 levels relative to β-tubulin, for which the data showed sufficient curvature for curve fitting by non-linear regression.

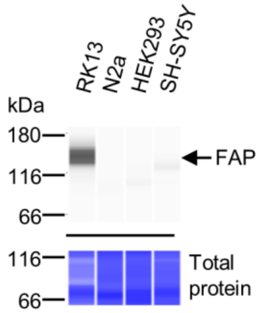

Fig. S3. Capillary western image showing lysates of several different cell lines assayed for FAP expression.

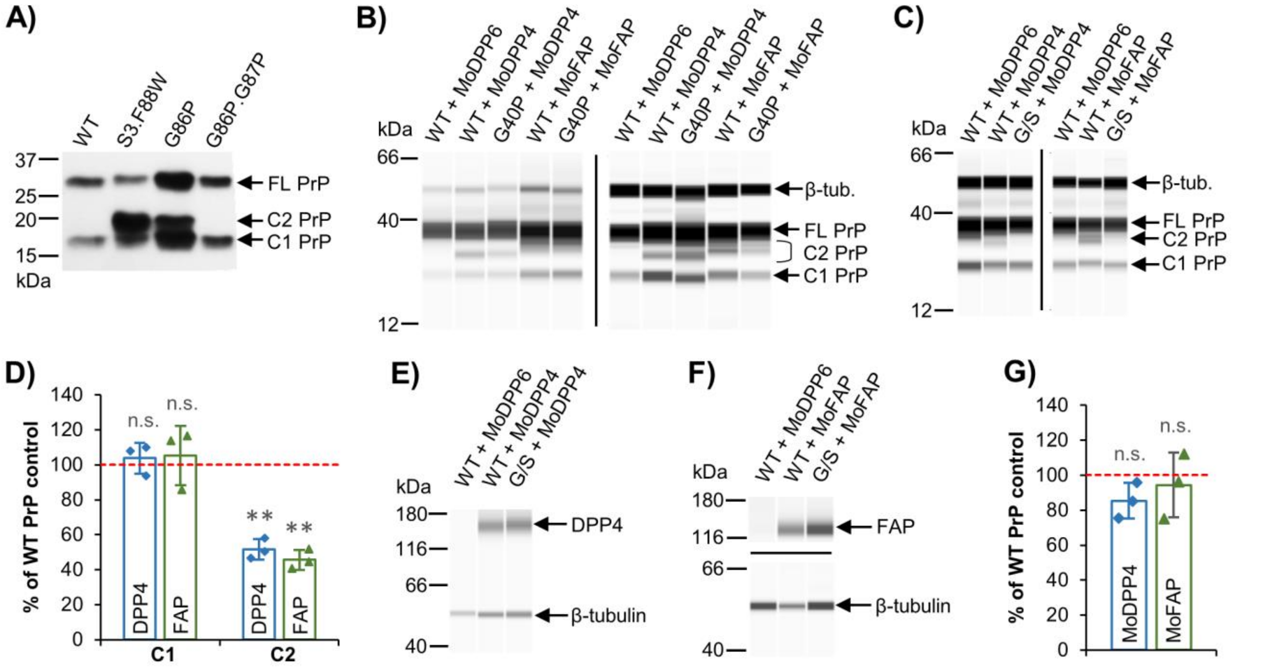

Fig. S4. Insights into DPP4 and FAP cleavage sites within PrP^{C} obtained using PrP muteins.
(A) Immunoblot (Sha31 antibody) showing PrP immunostaining in PNGase F-treated RK13 cell lysates obtained following transient expression of the indicated PrP variants. **(B)** Capillary western data (Sha31 antibody; +PNGase F) from RK13 cells transiently co-expressing WT or G40P PrP and the indicated S9B peptidases (the vertical line divides data from two independent experiments). **(C, E, F)** Representative capillary western images (+PNGase F for panel **(C)** only) and **(D, G)** charts showing that levels of C2 relative to total PrP in RK13 cells were lower when MoDPP4 or MoFAP were transiently co-expressed with G/S-switch PrP compared with WT MoPrP (one-sample t-tests; n = 3 independent experiments; **, $p < 0.01$), and that this effect was not due to differences in MoDPP4 or MoFAP expression levels. Data are shown as means ± S.D. Different contrast settings were applied to each part of **(C, F)**, as indicated by the dividing lines. WT MoPrP lanes in **(C)** display signals from the same capillaries as the equivalent lanes of Fig. 2A. The Sha31 anti-PrP antibody was used for obtaining the panel **(C)** image.

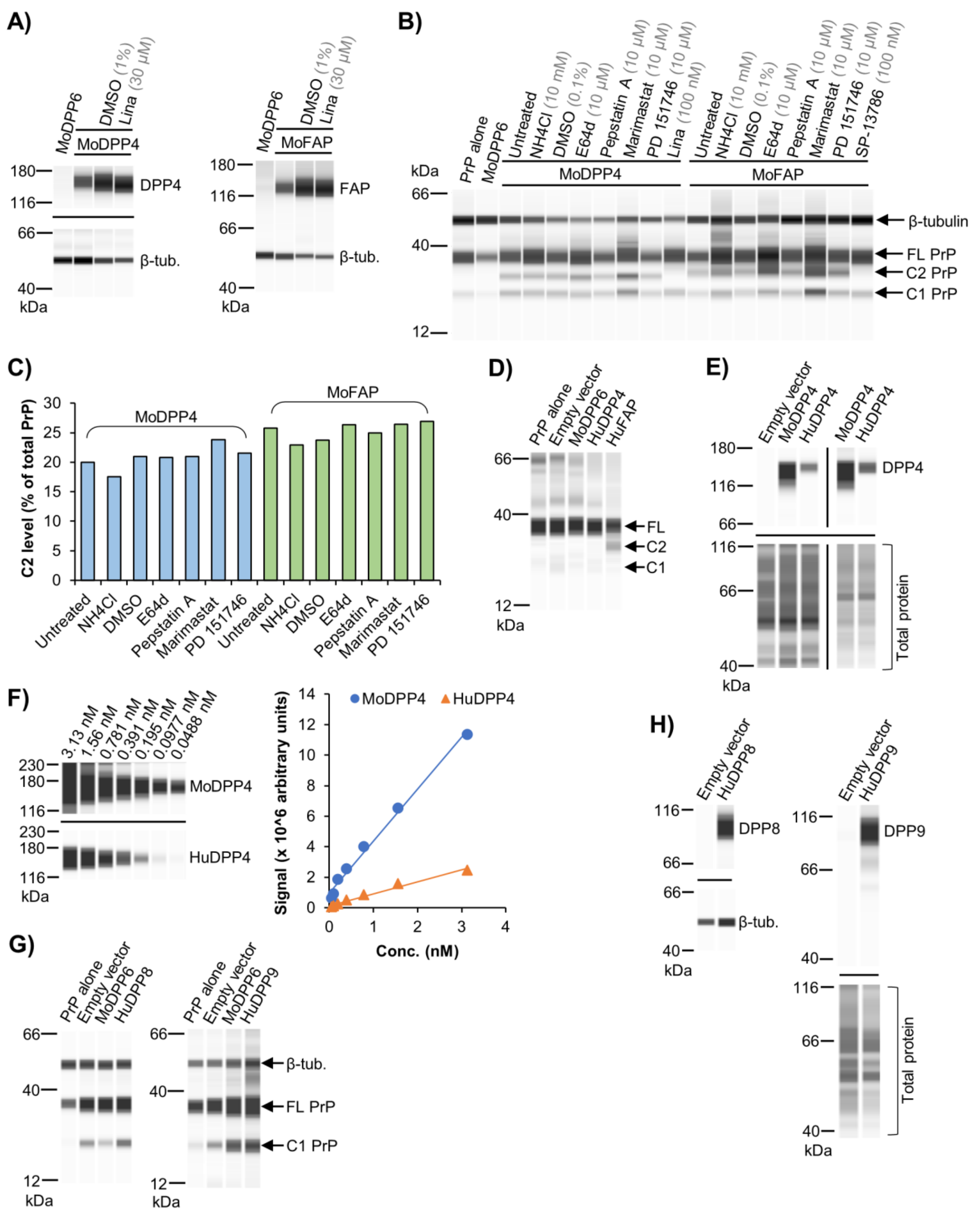

Fig. S5. Extended data from co-transfection experiments. **(A)** Representative capillary western data from RK13 cells transiently expressing WT MoPrP and the indicated S9B peptidases showing that DPP4 and FAP expression levels were similar irrespective of whether linagliptin was present or absent (n = 3 independent experiments). Different contrast settings were applied to each part of the left-hand image, as

indicated by the dividing line. **(B, C)** Capillary western image (Sha31 antibody; +PNGase F) and chart showing the effects of various protease inhibitors on C2 production induced by transient co-expression of WT MoPrP and MoDPP4 or MoFAP in RK13 cells. NH_4Cl inhibits lysosomal activity, E64d inhibits cysteine proteases, pepstatin A inhibits aspartic acid proteases, marimastat inhibits metalloproteases, and PD 151746 inhibits calpains. **(D)** Representative capillary western data (Sha31 antibody; +PNGase F) from HEK293T cells showing the effects of transient expression of the indicated S9B peptidases on fragmentation of the endogenous PrP^C expressed by these cells (n = 2 independent experiments). **(E)** Capillary western images showing the DPP4 signals obtained from HEK293T cells transfected with MoDPP4 or HuDPP4 expression vectors (the vertical line divides data from two independent experiments). The empty vector condition (pcDNA3) was the control for HuDPP4; an exact empty vector control for MoDPP4 was unavailable. The lower DPP4 signals in lysates of HuDPP4-transfected cells can be explained by the lower potency of the DPP4 antibody (R&D Systems, AF954) towards HuDPP4 compared with MoDPP4, as demonstrated in **(F)**, in which dose ranges of mouse and human recDPP4 were analysed. **(G)** Representative capillary western data (Sha31 antibody; +PNGase F) from HEK293T cells transiently expressing HuPrP and the indicated S9B peptidases showing that HuDPP8 and HuDPP9 do not induce β-cleavage (n = 2 independent experiments). **(H)** Capillary western images confirming that HuDPP8 and HuDPP9 were expressed efficiently in HEK293T cells co-transfected with HuPrP and HuDPP8 or HuDPP9 expression vectors. Different contrast settings were applied to each part of the left-hand image, as indicated by the dividing line.

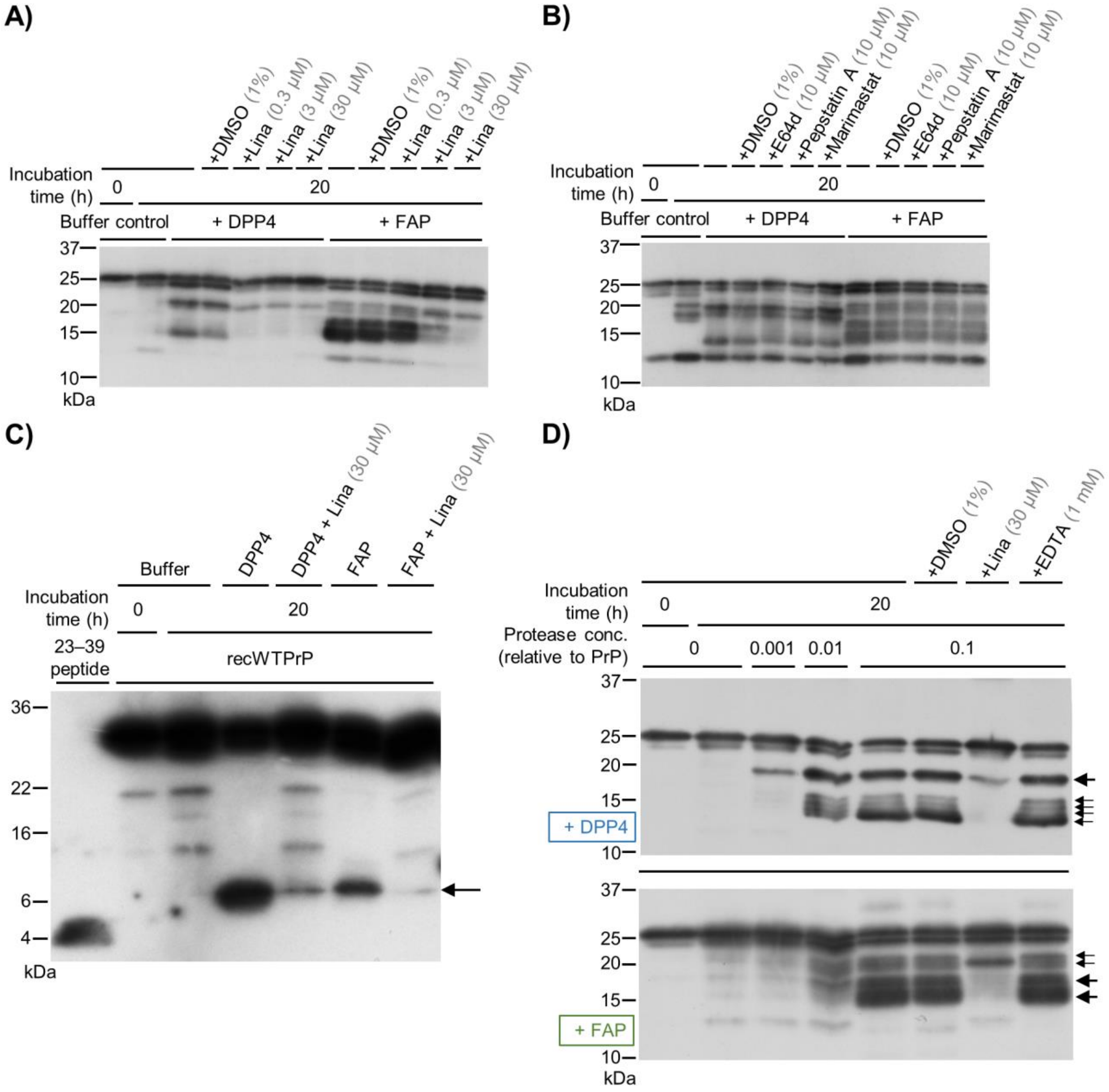

Fig. S6. Further analyses of recDPP4- and recFAP-generated recWTPrP fragments. **(A, B)** Immunoblots (Sha31 antibody) showing the effects of different protease inhibitors on recWTPrP fragmentation driven by incubation with recDPP4 or recFAP. **(C)** Immunoblot (EB8 antibody; epitope = 26–34) showing the presence of an N-terminal fragment (indicated by the arrow) following incubation of recWTPrP with recDPP4 or recFAP. A synthetic peptide (residues 23–39 of murine PrP) was included in case smaller fragments were detectable. **(D)** Immunoblot (12B2 antibody; epitope 88–92) showing the fragmentation patterns observed following incubation of recWTPrP with recDPP4 or recFAP. Fragments generated by the proteases are indicated by arrows. For all panels, the protease concentration was 1/10 of the PrP concentration.

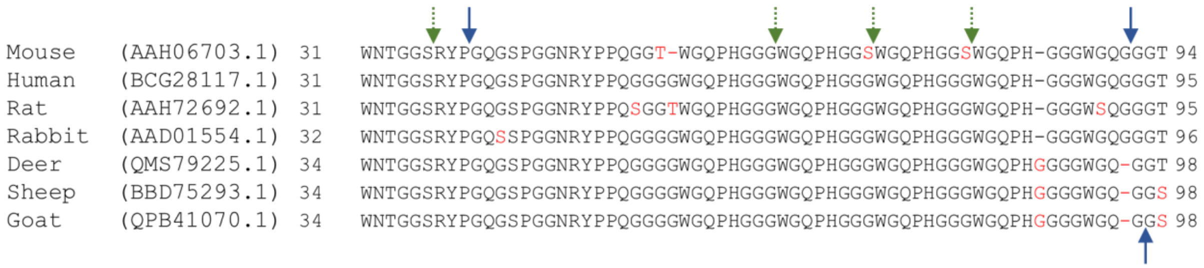

Fig. S7. Alignment of PrP^{C} sequences from several species. NCBI accession numbers are indicated and sequence differences versus human are marked in red. DPP4 and FAP cleavage sites identified for the mouse sequence are indicated. DPP4 cleavage sites are denoted by blue arrows, FAP cleavage sites by green arrows, and probable cleavage sites by dotted arrows.

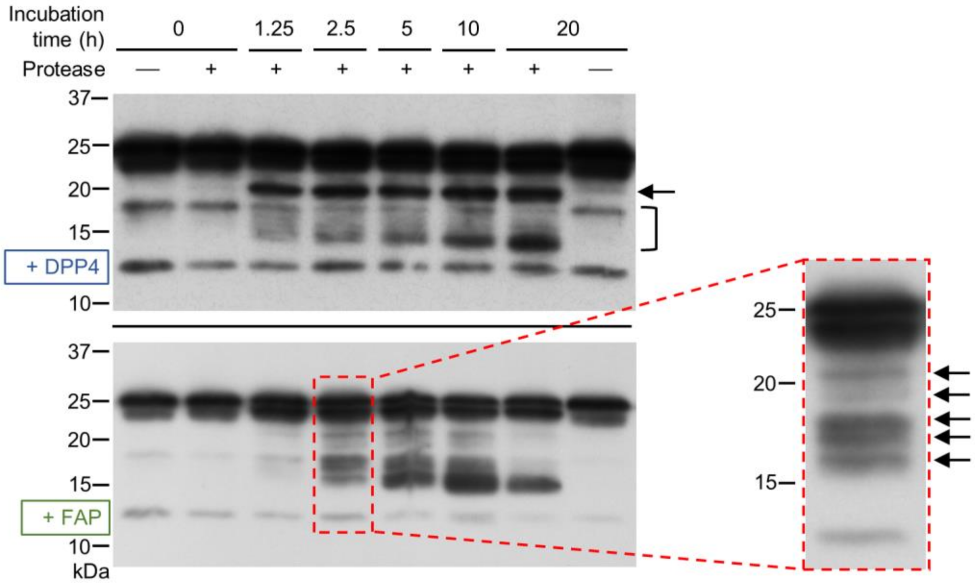

Fig. S8. Immunoblot (Sha31 antibody) showing the progression of recWTPrP fragmentation as incubation time with recDPP4 or recFAP increases (protease concentration was 1/10 of the PrP concentration). The higher magnification image is used to highlight the different bands observed.

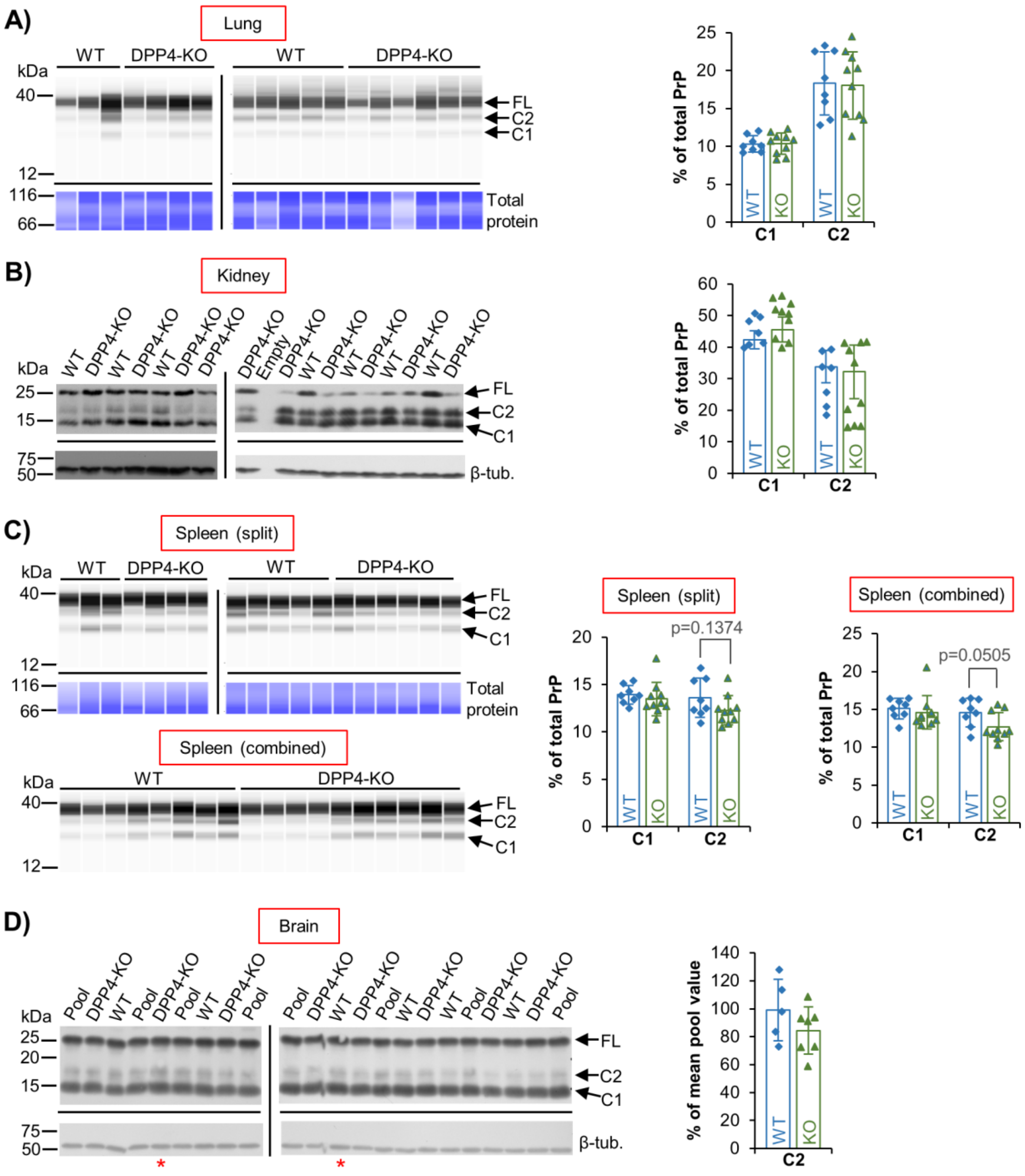

Fig. S9. Analysis of PrP^{C} fragmentation in various tissues from *Dpp4*-KO mice. **(A–D)** Relative C1 and C2 levels in PNGase F-treated homogenized mouse tissues were determined by capillary westerns **(A, C)** or conventional western blotting **(B, D)** using the Sha31 anti-PrP antibody. Conventional western blots were used for kidney and brain tissues because the somewhat lower sensitivity of the capillary western system prevented accurate quantification of PrP^{C} signals in general (kidney) or C2 signals specifically (brain). Data in the charts are shown as means ± S.D. No significant effect of *Dpp4* genotype on PrP^{C} fragmentation was detected in any tissue, as determined by unpaired two-sample t-tests (n = 5–10; $p > 0.05$). Vertical dividing lines indicate that samples were analysed on different capillary western plates or western blots. Panel **(C)** shows data from the same spleen homogenates analysed either in two batches (“split”) or in a single assay (“combined”). No total protein image could be obtained for the

“combined” analysis of spleen homogenates due to the limited number of capillaries that can be analysed simultaneously. For panel **(D)**, a pool of all the samples was included in quadruplicate on each blot to control for inter-blot variability; C2 signals relative to total PrP are expressed as percentages of the mean value obtained from the sample pool. Asterisks indicate samples that were not included in quantification due to air bubbles overlapping with bands.

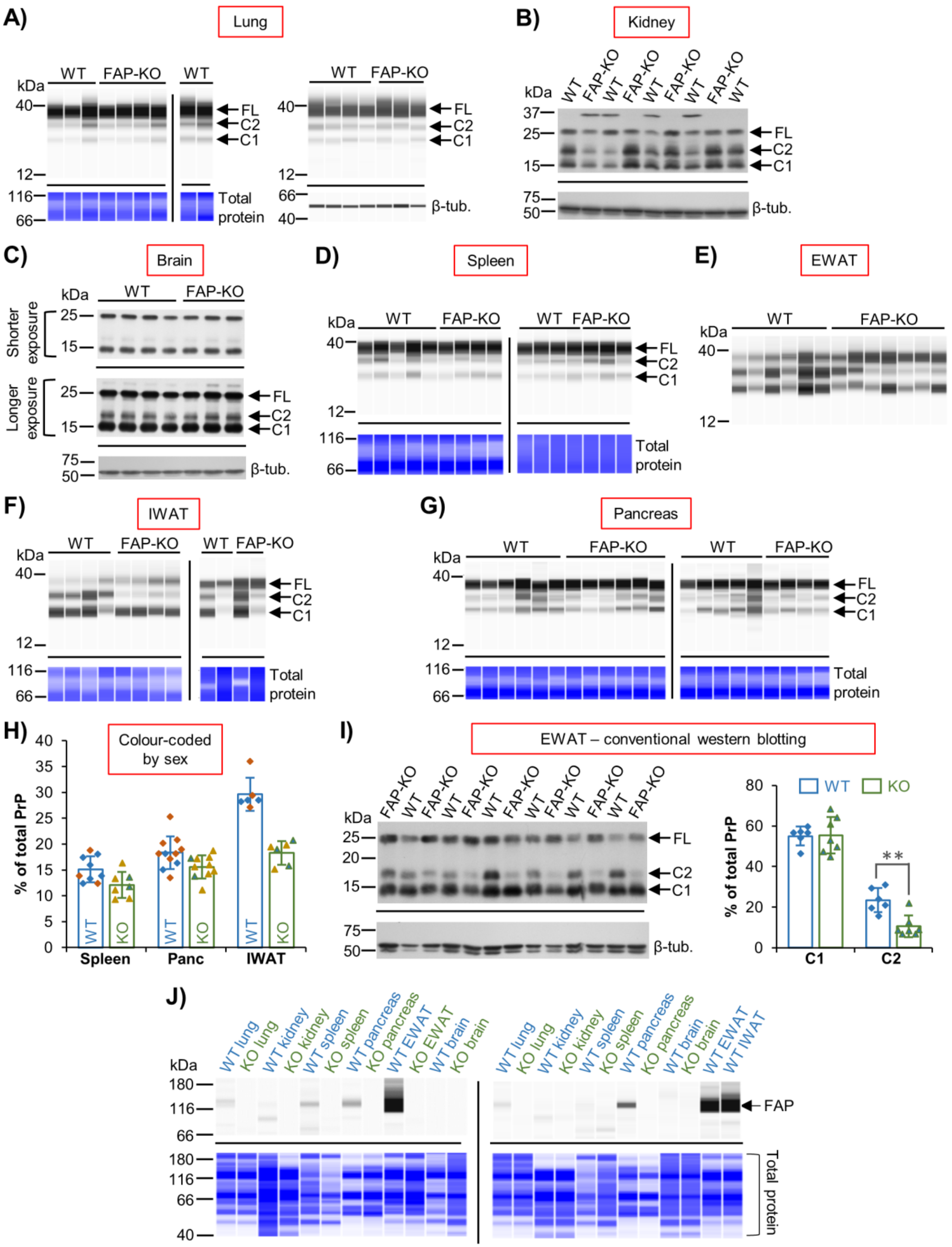

Fig. S10. Further data from analyses of PrP^{C} fragmentation in various tissues from *Fap*-null mice. **(A–G)** Relative C1 and C2 levels in PNGase F-treated homogenized mouse tissues were determined by capillary

westerns **(A, D–G)** or conventional western blotting **(B, C)** using the Sha31 anti-PrP antibody. The quantification data displayed in Fig. 5 derive from these images. Vertical dividing lines indicate that samples were analysed on different capillary western plates or western blots. Conventional western blots were used for kidney and brain tissues because the somewhat lower sensitivity of the capillary western system prevented accurate quantification of PrP^C signals in general (kidney) or C2 signals specifically (brain). In panel **(C)**, the C2 bands were quantified from the longer exposure and the FL and C1 bands from the shorter exposure. No total protein image could be obtained for EWAT **(E)** due to a recurring problem with electrophoretic separation specific to these samples. **(H)** Chart re-capitulating the C2 data from Figs. 5D, F and G, but with the sex of each sample marked (blue = WT, male; orange = WT, female; green = KO, male; gold = KO, female). EWAT data are not shown here since all EWAT samples were male. No sex-specific differences could be detected. **(I)** Confirmatory western blot of EWAT homogenates (Sha31 antibody; +PNGase F) and chart showing a significant *Fap* KO-induced reduction in C2 levels similar to that observed by capillary westerns. Data are shown as means ± S.D. (Mann-Whitney U test; $n \geq 6$; **, $p < 0.01$). **(J)** Capillary western data showing FAP expression levels in various WT and *Fap*-null mouse tissues. It was not possible for all tissues of each genotype to be from the same individual mouse, but two sets of tissues analysed in independent experiments (separated by the dividing line) gave similar results.

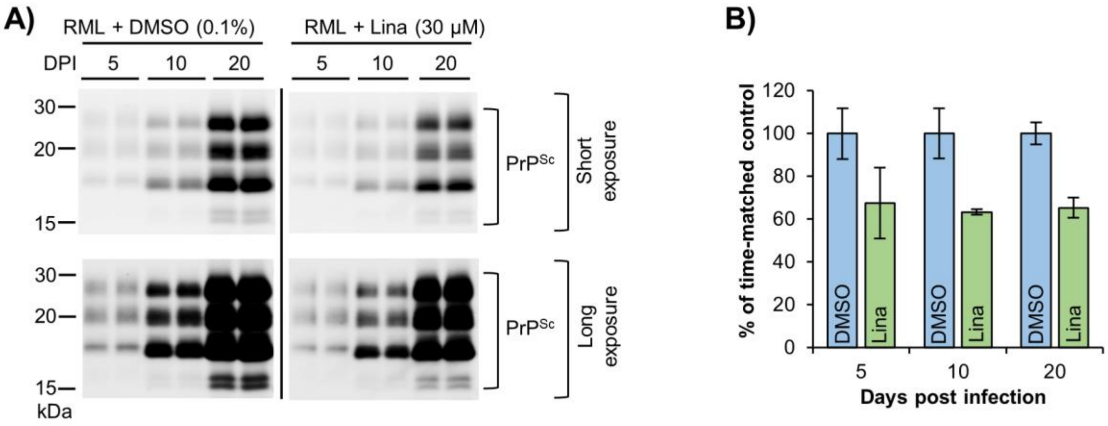

Fig. S11. Linagliptin treatment reduces PrP^{Sc} levels in prion-infected C2C12 cells. **(A)** Immunoblot (Sha31 antibody) and **(B)** chart showing the effects of linagliptin or vehicle (DMSO) control treatment on PrP^{Sc} levels in PK-treated lysates of RML-infected cells. Data are shown as means ± S.D. (n = 2 technical replicates at each time point). The longer exposure was used for quantification at the 5 DPI time point. To aid visual interpretation, the two halves of the blot have been swapped over from their original positions, as indicated by the vertical dividing line.

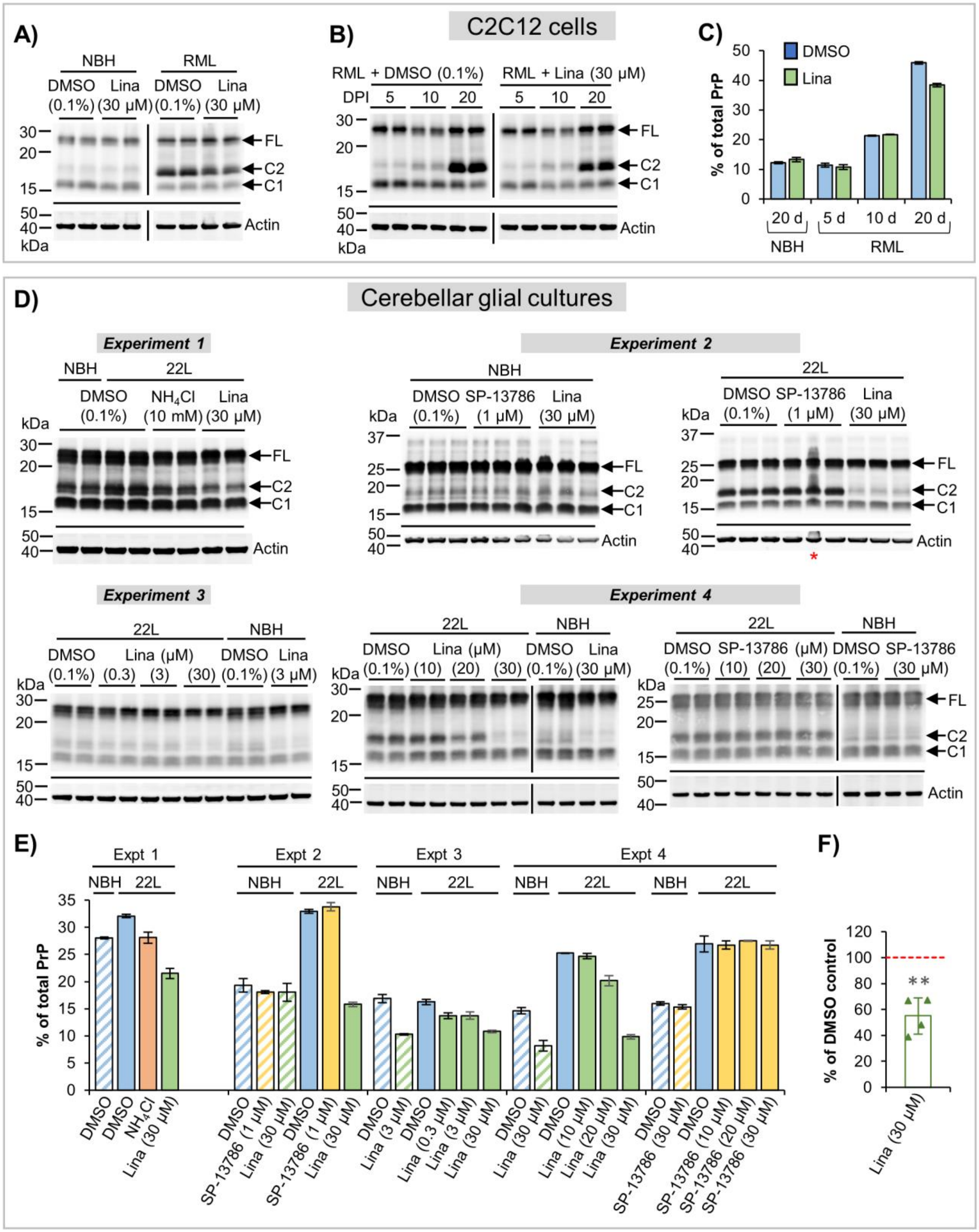

Fig. S12. Linagliptin treatment inhibits the increase in summed C2 levels observed in prion-infected cells. **(A, B, D)** Immunoblots (Sha31 antibody; +PNGase F) and **(C, E)** charts (means ± S.D.) showing the effects of different compound treatments on summed C2 levels relative to total PrP in two models of prion

infection (n = 2 technical replicates for RML prions in C2C12 cells and n = 4 independent experiments consisting of n ≥ 2 technical replicates for 22L prions in primary cerebellar glial cultures). DMSO was the vehicle control for all compound treatments. Lysates from cerebellar glial cultures were prepared either 21 DPI (experiments 1 and 2), 28 DPI (experiment 3) or 35 DPI (experiment 4). The 20 d time point samples shown in **(B)** were analysed on a separate immunoblot **(A)** to compare with NBH control samples from the same time point. RML 20 d time point data in **(C)** derive from panel **(A)** data, because the exposure was more amenable to accurate quantification. The vertical dividing line in **(A)** indicates that empty lanes have been removed. The vertical dividing line in **(B)** indicates that the two halves of the blot have been swapped over from their original positions to aid visual interpretation. The asterisk on panel **(D)** indicates that the sample in that lane was excluded from quantification due to abnormal SDS-PAGE migration. The vertical dividing lines in the Experiment 4 blots of panel **(D)** indicate that empty lanes have been removed and that the NBH lanes have been flipped horizontally to match the lane ordering for Experiment 3.
(F) Chart highlighting the consistent reduction in summed C2 levels relative to total PrP observed with 30 µM linagliptin treatment across all the experiments with 22L-infected cerebellar glial cultures (one-sample t-test; n = 4 independent experiments; **, $p < 0.01$).

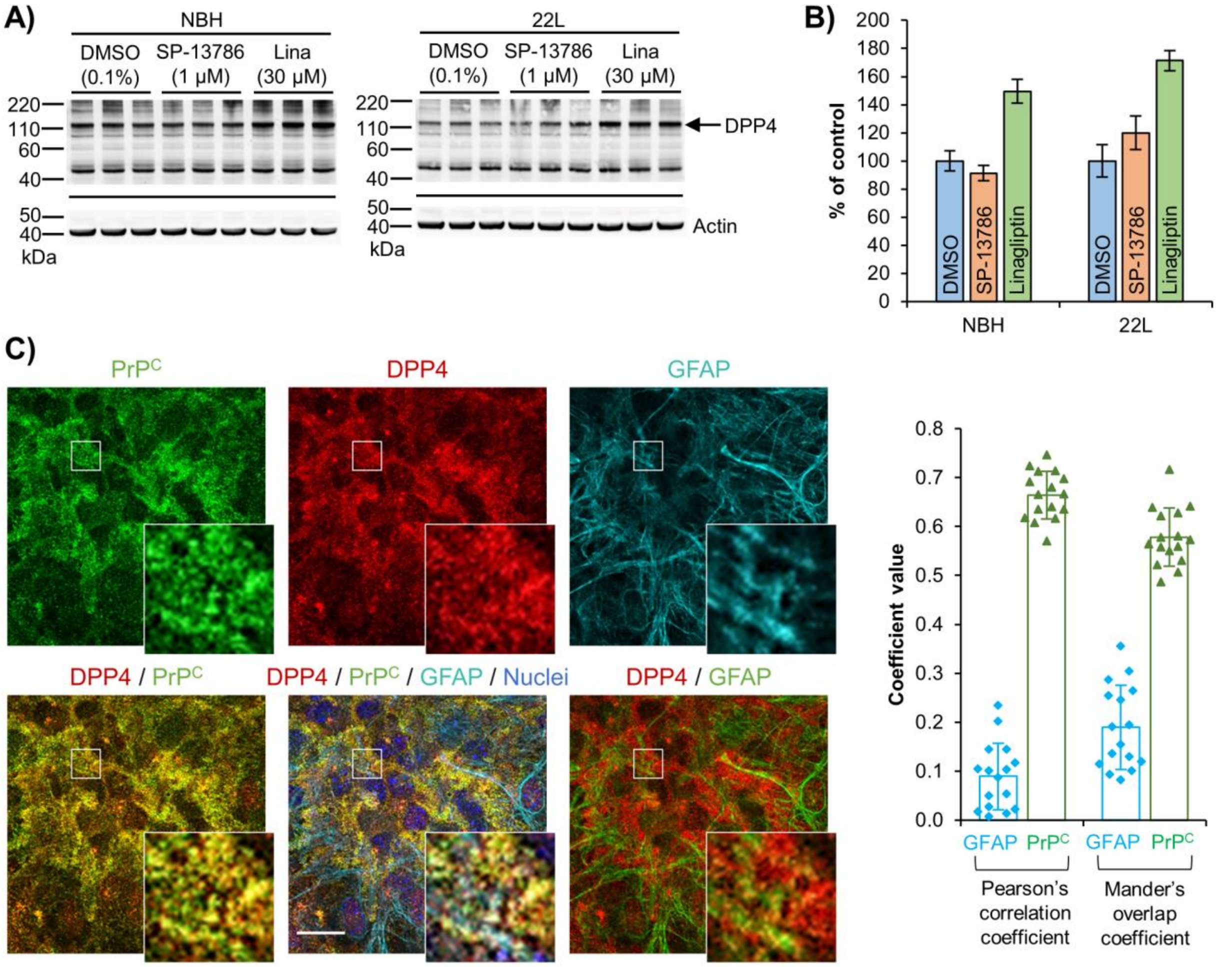

Fig. S13. Analysis of DPP4 expression in primary cerebellar glial cultures. **(A)** Immunoblots and **(B)** chart confirming that DPP4 is detected in the cultures and that its expression level may be increased by linagliptin treatment (n = 3 technical replicates). **(C)** Representative confocal images and chart showing co-localization of DPP4 and PrP^{C} immunostaining in (uninfected) primary cerebellar glial cultures. Data are shown as means ± S.D, with glial fibrillary acidic protein (GFAP) used as a control; co-localization of DPP4 with the astrocyte marker GFAP would not be expected because GFAP is expressed in the cytoplasm (n = 16 images from a single experiment). Scale bar = 20 μM.

Table S1. Mouse tissues analysed in this study.

Tissue	Genotype	Laboratory	# Males	# Females
Lung	*Dpp4* (+/+)	Ottawa (E.E.M.)	8	0
Lung	*Dpp4* (–/–)	Ottawa (E.E.M.)	10	0
Spleen	*Dpp4* (+/+)	Ottawa (E.E.M.)	8	0
Spleen	*Dpp4* (–/–)	Ottawa (E.E.M.)	10	0
Kidney	*Dpp4* (+/+)	Ottawa (E.E.M.)	8	0
Kidney	*Dpp4* (–/–)	Ottawa (E.E.M.)	10	0
Brain	*Dpp4* (+/+)	Ottawa (E.E.M.)	6	0
Brain	*Dpp4* (–/–)	Ottawa (E.E.M.)	6	2
Lung	*Fap* (+/+)	Ottawa (E.E.M.)	6	3
Lung	*Fap* (–/–)	Ottawa (E.E.M.)	2	5
Spleen	*Fap* (+/+)	Ottawa (E.E.M.)	6	3
Spleen	*Fap* (–/–)	Ottawa (E.E.M.)	2	5
Kidney	*Fap* (+/+)	Ottawa (E.E.M.)	3	2
Kidney	*Fap* (–/–)	Ottawa (E.E.M.)	1	3
Brain	*Fap* (+/+)	Ottawa (E.E.M.)	3	1
Brain	*Fap* (–/–)	Ottawa (E.E.M.)	1	2
Pancreas	*Fap* (+/+)	Ottawa (E.E.M.)	3	2
Pancreas	*Fap* (–/–)	Ottawa (E.E.M.)	1	3
Pancreas	*Fap* (+/+)	Toronto (D.J.D.)	0	6
Pancreas	*Fap* (–/–)	Toronto (D.J.D.)	1	5
EWAT	*Fap* (+/+)	Toronto (D.J.D.)	6	0
EWAT	*Fap* (–/–)	Toronto (D.J.D.)	7	0
IWAT	*Fap* (+/+)	Toronto (D.J.D.)	3	3
IWAT	*Fap* (–/–)	Toronto (D.J.D.)	3	3

Table S2. Manufacturer/supplier details for resources used in this study

Resource	Manufacturer/supplier	Part number
Cell lines		
C2C12 (female)	American Type Culture Collection	CRL-1772
RK13 (sex not reported)	American Type Culture Collection	CCL-37
HEK293 (female)	American Type Culture Collection	CRL-1573
HEK293T (female)	American Type Culture Collection	CRL-3216
Reagents for cell experiments		
DMEM (low-glucose)	Gibco	LS11885084
MEM	MilliporeSigma	M4655
FBS	Life Technologies	LS12483020
Horse serum	Gibco	LS16050122
Pen-strep solution	Gibco	LS15140122
Poly-D-lysine hydrobromide	MilliporeSigma	P6407
Lipofectamine 3000 kit	Invitrogen	L3000008
Plasmid vectors		
pCMV-Sport6.moDPP4	Open Biosystems	N/A[1]
pCMV-Sport6.moFAP	Horizon Discovery	MMM1013-202767860
pcDNA3.1.huDPP4	Genscript	OHu23624
pcDNA3.1.huDPP8	Genscript	OHu05868
pcDNA3.1.huDPP9	Genscript	OHu06272D
pcDNA3.1.huFAP	Genscript	OHu27944
Primary antibodies		
Sha31 anti-PrP	Bertin Bioreagent	A03213
12B2 anti-PrP	Gift from Dr. Jan Langeveld	N/A
EB8 anti-PrP	Gift from Dr. Giuseppe Legname	N/A
Anti-GFAP	Abcam	ab4674
Anti-β-tubulin	Novus Biologicals	NB600-936
Anti-DPP4 (used for capillary westerns)	R&D Systems	AF954
Anti-DPP4 (used for conventional western blots and immunofluorescence staining)	Abcam	ab187048
Anti-FAP	Abcam	ab207178
Anti-DPP8	Novus Biologicals	NBP2-01830
Anti-DPP9	Novus Biologicals	NBP2-01521
Anti-β-actin	Sigma	A5441
Capillary western reagents		
12–230 kDa Separation Module	ProteinSimple	SM-W004
Anti-Mouse Detection Module	ProteinSimple	DM-002
Anti-Rabbit Detection Module	ProteinSimple	DM-001
Anti-Goat Detection Module	ProteinSimple	DM-006
Total Protein Detection Module	ProteinSimple	DM-TP01
Protein Normalization Module	ProteinSimple	DM-PN02
Recombinant proteins		
Recombinant wild-type PrP	Gift from Dr. Valerie Sim	N/A
Recombinant S3 PrP	Gift from Dr. Nathalie Daude	N/A
PrP23–39 peptide	Gift from Dr. Oliver Julien	N/A

Recombinant mouse DPP4	Novus Biologicals	954-SE-010
Recombinant mouse DPP4	Sino Biological	50718-M01H-10
Recombinant human DPP4	Novus Biologicals	9168-SE-010
Recombinant mouse FAP	Novus Biologicals	8647-SE-010
Instrumentation		
LSM700 confocal microscope	Zeiss	N/A
Jess capillary western machine	ProteinSimple	N/A
Wes capillary western machine	ProteinSimple	N/A
Miscellaneous		
6-well tissue culture-treated microplates	Falcon	C353046
96-well tissue culture-treated microplates	Falcon	C353072
96-well untreated microplates	Greiner Bio-One	655201
Amicon Ultra-15 Centrifugal Filter Units	MilliporeSigma	UFC900396
Bicinchoninic acid assay kit	Thermo Scientific	0023225
Complete protease inhibitor cocktail	Roche	04693159001
Coomassie Brilliant Blue R-250	Bio-Rad	1610400
DiscoveryProbe Protease Inhibitor Library[2]	ApexBio,	L1035
EndoFree Plasmid Maxi Kit	Qiagen	12362
Gibson Assembly Cloning Kit	New England Biolabs	E5510S
Hoechst 33342	Invitrogen	H1399
Immobilon-PSQ polyvinylidene fluoride membranes	MilliporeSigma	ISEQ00010
Linagliptin	Toronto Research Chemicals	L465900
Microscope cover glass	Fisherbrand	15CIR1602811G
N2a cell lysate	Gift from Dr. Satyabrata Kar	N/A
PBS	Boston Bioproducts	BM-220
PNGase F kit	New England Biolabs	P07040S
SH-SY5Y cell lysate	Gift from Dr. Satyabrata Kar	N/A
SP-13786	SelleckChem	S0842

[1] Open Biosystems reagents are now sold by Horizon Discovery but this vector was purchased before that change occurred.
[2] Used for the initial compound screen as well as being the source of E64d, pepstatin A, marimastat and PD 151746 protease inhibitors used in later experiments.

Dataset S1 (separate file). Protease inhibitor library screen dataset. Capillary western quantification data (Sha31 antibody) are provided for the 130 protease inhibitors that were tested in S3-3 RK13 cells (20 µM for 4 d) to identify modulators of S3 PrP fragmentation. Fold changes are expressed in comparison to the mean value from the vehicle controls (1% [v/v] DMSO). Grey text indicates compounds considered toxic (those that reduced β-tubulin signals by >2-fold).
[1]Heat shock protein 90 is not a protease itself but is required for maintaining proteasome structure.
[2]Target is not a protease.
[3]Compound activates a protease rather than inhibits it.

SI References

1. A. R. Castle, N. Daude, S. Gilch, D. Westaway, Application of high-throughput, capillary-based Western analysis to modulated cleavage of the cellular prion protein. *Journal of Biological Chemistry* 294, 2642-2650 (2019).

2. A. Lau *et al.*, Octarepeat region flexibility impacts prion function, endoproteolysis and disease manifestation. *Embo Molecular Medicine* 7, 339-356 (2015).

3. R. C. C. Mercer *et al.*, The Prion Protein Modulates A-type K+ Currents Mediated by Kv4.2 Complexes through Dipeptidyl Aminopeptidase-like Protein 6. *Journal of Biological Chemistry* 288, 37241-37255 (2013).

4. B. Drisaldi *et al.*, Genetic mapping of activity determinants within cellular prion proteins - N-terminal modules in PrPC offset pro-apoptotic activity of the Doppel helix B/B ' region. *Journal of Biological Chemistry* 279, 55443-55454 (2004).

5. D. Marguet *et al.*, Enhanced insulin secretion and improved glucose tolerance in mice lacking CD26. *Proceedings of the National Academy of Sciences of the United States of America* 97, 6874-6879 (2000).

6. B. L. Panaro *et al.*, Fibroblast activation protein is dispensable for control of glucose homeostasis and body weight in mice. *Molecular Metabolism* 19, 65-74 (2019).

7. J. P. M. Langeveld *et al.*, Rapid and discriminatory diagnosis of scrapie and BSE in retro-pharyngeal lymph nodes of sheep. *BMC veterinary research* 2, 19 (2006).

8. A. Didonna *et al.*, Characterization of four new monoclonal antibodies against the distal N-terminal region of PrPC. *Peerj* 3 (2015).

9. C. Feraudet *et al.*, Screening of 145 anti-PrP monoclonal antibodies for their capacity to inhibit PrPSc replication in infected cells. *Journal of Biological Chemistry* 280, 11247-11258 (2005).
